# P2X7 receptor inhibition ameliorates ubiquitin–proteasome system dysfunction associated with Alzheimer’s disease

**DOI:** 10.1186/s13195-023-01258-x

**Published:** 2023-06-07

**Authors:** Carolina Bianchi, Beatriz Alvarez-Castelao, Álvaro Sebastián-Serrano, Caterina Di Lauro, Lucia Soria-Tobar, Annette Nicke, Tobias Engel, Miguel Díaz-Hernández

**Affiliations:** 1 Department of Biochemistry and Molecular Biology, Veterinary School, Complutense University of Madrid, Avda. Puerta de Hierro S/N, 28040 Madrid, Spain; 2 Instituto de Investigación Sanitaria del Hospital Clínico San Carlos, IdISSC, Madrid, Spain; 3 Walther Straub Institute of Pharmacology and Toxicology, Faculty of Medicine, Ludwig-Maximilians-Universität München, Munich, Germany; 4 Department of Physiology and Medical Physics, RCSI, University of Medicine and Health Sciences, Dublin, D02 YN77 Ireland; 5 FutureNeuro, SFI Research Centre for Chronic and Rare Neurological Diseases, RCSI, University of Medicine and Health Sciences, Dublin, D02 YN77 Ireland

**Keywords:** Alzheimer’s disease, P2X7 receptor, Ubiquitin–proteasome system

## Abstract

**Background:**

Over recent years, increasing evidence suggests a causal relationship between neurofibrillary tangles (NFTs) formation, the main histopathological hallmark of tauopathies, including Alzheimer’s disease (AD), and the ubiquitin–proteasome system (UPS) dysfunction detected in these patients. Nevertheless, the mechanisms underlying UPS failure and the factors involved remain poorly understood. Given that AD and tauopathies are associated with chronic neuroinflammation, here, we explore if ATP, one of the danger-associated molecules patterns (DAMPs) associated with neuroinflammation, impacts on AD-associated UPS dysfunction.

**Methods:**

To evaluate if ATP may modulate the UPS via its selective P2X7 receptor, we combined in vitro and in vivo approaches using both pharmacological and genetic tools. We analyze postmortem samples from human AD patients and P301S mice, a mouse model that mimics pathology observed in AD patients, and those from the new transgenic mouse lines generated, such as P301S mice expressing the UPS reporter Ub^G76V^-YFP or P301S deficient of P2X7R.

**Results:**

We describe for the first time that extracellular ATP-induced activation of the purinergic P2X7 receptor (P2X7R) downregulates the transcription of β5 and β1 proteasomal catalytic subunits via the PI3K/Akt/GSK3/Nfr2 pathway, leading to their deficient assembly into the 20S core proteasomal complex, resulting in a reduced proteasomal chymotrypsin-like and postglutamyl-like activities. Using UPS-reported mice (UbGFP mice), we identified neurons and microglial cells as the most sensitive cell linages to a P2X7R-mediated UPS regulation. In vivo pharmacological or genetic P2X7R blockade reverted the proteasomal impairment developed by P301S mice, which mimics that were detected in AD patients. Finally, the generation of P301S;UbGFP mice allowed us to identify those hippocampal cells more sensitive to UPS impairment and demonstrate that the pharmacological or genetic blockade of P2X7R promotes their survival.

**Conclusions:**

Our work demonstrates the sustained and aberrant activation of P2X7R caused by Tau-induced neuroinflammation contributes to the UPS dysfunction and subsequent neuronal death associated with AD, especially in the hippocampus.

**Supplementary Information:**

The online version contains supplementary material available at 10.1186/s13195-023-01258-x.

## Background

The ubiquitin–proteasome system (UPS) is the mayor pathway for protein degradation in eukaryotic cells in a specific and selective way. After the covalent attachment of multiple ubiquitin molecules, most target proteins are degraded in an ATP-dependent way via the 26S proteasome complex [1, 2]. The 26S proteasome complex is composed of two subcomplexes: the 19S regulatory cap that recognizes, deubiquitinates, unfolds, and translocates substrates into the 20S complex where these are digested into small peptides [2]. Several pathological conditions may impact on the UPS, thereby, affecting its functionality [3–6]. In neurodegenerative diseases, UPS dysfunction may promote the accumulation of aberrant intracellular protein inclusions thereby contributing to disease pathogenesis [7, 8]. In Alzheimer’s disease (AD), the most common human neurodegenerative disorder, aberrant intracellular aggregates, called neurofibrillary tangles (NFTs), are assembled by hyperphosphorylated microtubule-associated protein tau [9]. The fact that the UPS degrades tau protein [10, 11] and that a pharmacological proteasome inhibition favors the aggregation of tau [12] supports the idea that there is a causal relationship between NFTs formation and UPS dysfunction detected in AD patients. Accordingly, it has been shown that AD patients present a decreased proteasomal activity in severely degenerated brain areas but not in less or unaffected regions [13, 14]. These patients also show an accumulation of polyubiquitinated proteins [15] and recruitment of various UPS elements in NTFs [12, 16, 17]. Considering the late onset of AD-associated symptoms, it has been postulated that a reduced UPS activity due to aging may be one of the triggering factors of AD. However, this would not explain why not all elderly people develop AD or why specific brain regions and/or certain types of neural cells are more prone than others to develop intracellular aggregates. To date, what causes UPS dysfunction in AD remains unknown.

Over recent years, increasing evidence suggests the involvement of the ATP-sensitive plasma membrane receptors, P2 receptors, in different neurodegenerative disorders [18]. P2 receptors are divided into two subgroups: ionotropic P2X and metabotropic P2Y receptors. Currently, eight P2Y subunits (P2Y1,2,4,6,11,12,13,14) and seven P2X subunits (from P2X1 to P2X7) have been cloned and characterized in human or other mammals [19], which assemble to form functional homomeric or heteromeric receptors. Altogether, they can respond to a wide range of extracellular ATP concentrations (from nanomolar to millimolar range) [20], being the homomeric P2X7 receptor (P2X7R) is the one with the lowest ATP affinity. Specifically, related to AD, it has been reported that (i) both brains of AD patients [21–23] and those of P301S mice, a mouse model overexpressing mutated tau protein and reproduces the AD-associated Tauopathy, present elevated P2X7R expression levels [24–27], (ii) P2X7R is a major driver of neuroinflammation [21, 22], and (iii) is involved in neuronal death [28, 29] associated with AD. Previous studies reported that extracellular ATP and other extracellular adenosine nucleotides may regulate proteasome activity [30] and contribute to UPS impairment associated with neuroinflammation [4] via their selective plasma membrane receptors. Therefore, we decided to evaluate if altered P2X7R signaling contributes to an AD-associated UPS impairment. Herein, we demonstrate, for the first time, that P2X7R activation reduces proteasome activity by reducing the transcription of two catalytic proteasomal subunits β1 and β5 via the Akt kinase/glycogen synthase kinase 3 (GSK3)/nuclear factor erythroid 2-related factor 2 (Nfr2) pathway. Furthermore, we found in the hippocampus of both human AD patients and P301S mice a correlation of P2X7R upregulation with a decrease in the catalytic activity of the proteasome. Finally, in good agreement, we show that a genetic ablation of the P2X7R or its pharmacological inhibition prevents an UPS impairment and associated neuronal death in P301S mice.

## Methods

### Human brain tissues

Human samples were provided by the Banco de Tejidos Fundación CIEN (BT-CIEN, Madrid, Spain). A written informed consent for brain removal after death for diagnostic and research purposes was obtained from the brain donors and/or next of kin. Procedures, information, and consent forms were approved by the Bioethics Subcommittee of Fundación Cien Madrid, Spain (S19001). Supplementary Table 1 summarizes the gender and age of the patients with AD or control heathy from whom the hippocampal post-mortem samples were collected.

### Animals

All animal procedures were carried out at the Universidad Complutense de Madrid (UCM), in compliance with National and European regulations (RD1201/2005; 86/609/CEE) and following the guidelines of the International Council for the Laboratory Animal Science. The protocol for animal experiments was approved by the Committee of Animal Experiments at the UCM and the Environmental Counselling of the Comunidad de Madrid, Spain (PROEX 374/15 and PROEX 185/17).

Mice expressing UbGFP [line B6.Cg-Tg(CAG-Ub*G76V/GFP)2Dant/J] were provided by Dr. J.J Lucas (CBMSO, Madrid, Spain). The P301S mice were obtained from Jackson laboratory [line B6; C3-Tg (Prnp-MAPT*P301S) PS19Vle/J; stock number 008169]. The original B6C3H/F1 genetic background was changed to C57BL/6 J by back-crossing them with C57 animals in our laboratory. P2X7R null mice (P2X7^−/−^) [line B6N-P2rx7tm1d(EUCOMM)Wtsi/Ieg)] were provided by Prof. Annette Nicke. Heterozygous P301S mice (P301S) were crossed with P2X7^−/−^ mice, and subsequently back-crossed to finally obtain double P301S; P2X7^−/−^. P301S^+/−^ mice (P301S) were crossed with UbGFP mice to obtain P301S;UbGFP mice.

Animals were housed in a light and temperature-controlled humid environment, with a 12 h light–dark cycle and a light onset at 08:00 am. The mice were grouped 4–6 per cage and allowed free access to water and food (ad libitum). All surgeries were performed under isoflurane anesthesia, and all efforts were made to minimize suffering. Investigators were blinded to the group allocation during the animal experiments.

### PCR genotyping

Genomic DNA was obtained from tail biopsies using Wizard® SV Genomic DNA Purification System (Promega, Madison, WI, USA) according to the manufacturer’s protocol.

Simple PCR reactions were carried out using: for P301S mice DNA Kapa2G Fast HS Master Mix from Sigma-Aldrich (Madrid, Spain), specific primers (0.5 μM each) and 2 μL of genomic DNA in a final volume of 12 μL; for Ub^G76V^-GFP mice DNA Amplitools Master Mix (Biotools, Madrid, Spain), specific primers (10 μM each) and 2 μL of genomic DNA in a final volume of 24 μL; for Ub^G76V^-GFP;P2X7^−/−^ mice Econo PLUS Green 2 × Master Mix, specific primers (10 μM each) and 1 μL of genomic DNA in a final volume of 25 μL.

Animals were genotyped using specific primers for *P301S* Fw 5′-GGCATCTCAGCAATG TCTCC-3′ and Rv 5′-GGTATTAGCCTATGGGGGACAC-3′; for *P2rx7*^*−/−*^ Fw 5′-CTGGCAACTATCCATTTTCC-3′ and Rv 5′-GTGTGAGTGAATGAGATCGTG-3′; for *Ub*^*G76V*^*-GFP* Fw 5′-CCTACGGCGTGCAGTGCTTCAGC-3′ and Rv 5′-CGGCGAGCTGCACGCTGCGTCCTC-3′. Amplification was carried out in a Gene Amp2400 thermal cycler (Applied Biosystems). PCR amplification products were electrophoresed on a 1.5% (w/v) agarose gel and stained with SYBR® Safe DNA Gel Stain (Life Technologies CA, USA). PCR bands were visualized by gel imaging system Gel Logic 200 Imaging System (Kodak, Rochester, NY, USA).

### Intracerebral administration of drugs

Six‐month‐old WT or P2X7^−/−^ mice were anesthetized with isoflurane (1‐chloro‐2, 2, 2‐trifluoroethyl‐difluormethylether; Isovet; Braun, Rubi, Barcelona, Spain), diluted in 50% O_2_. Mice were kept under anesthesia during the surgery. The scalp was incised along the midline, and 1 hole was made at the appropriate stereotaxic coordinates from bregma (mediolateral, 1 mm; anteroposterior, 0.5 mm; dorsoventral, 2.5 mm). Intracerebroventricular administration of 2 μl of 30 mM BzATP or 2 μl of phosphate-buffered saline (PBS) was infused at a rate of ≈ 1 μl/min. After the injection, the needle was kept for an additional 2 min and then slowly withdrawn. Mice were euthanized 24 h after surgery and the hippocampus was micro-dissected for biochemical analysis.

### Intraperitoneal administration of drugs

P2X7 specific negative allosteric antagonist GSK1482160A was diluted at 10 mg/ml in vehicle solution. Vehicle solution was calcium- and magnesium-free sterile PBSplus 20% hydroxypropyl-β-cyclodextrin and 0.2% DMSO. 10 μL of GSK solution corresponding to a dosage of 100 mg per kg of body weight or the same volume of vehicle solution were daily intraperitoneally injected to 9-month-old P301S and WT mice per 3 weeks. The treatment protocol followed a single-blind design, by which the experimenter was unaware of the genotype and treatment applied to each mouse (*n* = 5–7 mice per group and condition). After treatment, mice were sacrificed, and brain tissue was processed to perform immunoblot and immunohistochemical analyses.

### Cell culture, transfection, and treatment conditions

Murine neuroblastoma cell line N2a was plated at 7.5 × 10^5^ cells/well in six-well plates and cultured in DMEM (Sigma-Aldrich) supplemented with Glutamax, 100 U/ml penicillin, 100 g/ml streptomycin, and 10% heat-inactivated fetal bovine serum (all from Gibco). Cells were grown at 37 °C in humidified atmosphere containing 5% CO_2_. 24 h after cells plated, when required, cells were transfected using Lipofectamine ™ 2000 (Invitrogen) following manufacturer’s instructions. Plasmid encoding Ub^G76V^-YFP, GFP-CL1 and ubiquitin-M-yellow fluorescent protein were provided by J.J.L. Commercial plasmid for overexpressing (*psmb5 and psmb1*) with the flag c-myc were provided by Origene (Rockville, Maryland, USA). Plasmids for overexpressing and silencing P2X7R were generated as previously described [31]. 24 h after transfection and before cells were stimulated with different drugs for the indicated periods, serum withdrawal to arrest the cell cycle. The selective inhibitors for the different kinases and the purinergic receptor antagonist assayed were pre-incubated 20 min before the addition of P2X7R agonist, the synthetic analog 2′(3′)-O-(4-benzoylbenzoyl)-ATP (BzATP).

### Cell viability assays

Twenty-four hours after the corresponding treatments, cell viability was tested by the MTT assay following the manufacturer’s instructions (Sigma-Aldrich). Values were normalized in respect to that obtained from untreated cells, considered as 100% survival.

### RNA extraction and quantitative real-time PCR (qRT-PCR)

Total RNA was extracted from hippocampi from human or adult mouse brains or from cultured N2a cells using a Speedtools total RNA Extraction Kit (Biotools), following manufacturer’s instructions. The animals were quickly sacrificed, and the hippocampi were immediately dissected and frozen using dry ice to proceed with total RNA isolation. After digestion with TURBO DNase (Ambion), 1 μg of total RNA was reverse transcribed with 6 μg of random primers, 350 μM dNTPs and M-MLV reverse transcriptase (all from Invitrogen). RT-PCRs were carried out using LuminoCt qPCR readymix (Sigma), 5 μl of the reverse transcribed product, and gene-specific primers and TaqMan MGB probes for Ub-GFP (Fw: 5′-TGCACCTGGTACTCCGTCT-3′; Rv: 5′-TCCAGCTCGACCAGGATG-3′), *GAPDH* (Fw: 5′-CACCACCAACTGCTTAGCCC-3′; Rv: 5′-TGTGGTCATGAGCCCTTCC-3′) (Applied Biosystems), *pmsb5* (Fw: 5′- ATCGAAATGCTTCACGGAAC-3′; Rv: 5′- CGTTCCTTATTGCGAAGCTC-3′) and *pmsb6* (Fw: 5′- CTGGGAAAACCGGGAAGTCT-3′;Rv: 5′- GGTTGTCCTGGAGTCCGCT-3′) (Sigma), *pmsb7* (Fw: 5′-TGCCTTATGTCACCATGGGTT-3′; Rv: 5′- CTTGGCTTCTTCCTCCTCCA-3′) (Sigma), *PSMB5* (Fw: 5′- CCATACCTGCTAGGCACCAT-3′; Rv: 5′- GCACCTCCTGAGTAGGCATC-3′), *PSMB6* (Fw: 5′- CTGGGAAAACCGGGAAGTCT-3′;Rv: 5′- GGTTGTCCTGGAGTCCGCT-3′).

Fast thermal cycling was performed using a StepOnePLus Real-Time PCR System (Applied Biosystems) as follows: denaturation, one cycle of 95 °C for 20 s, followed by 40 cycles each of 95 °C for 1 s and 60 °C for 20 s. The results were normalized as indicated by the parallel amplification of the endogenous control glyceraldehyde-3-phosphate dehydrogenase (GAPDH).

### Proteasome activity assays

N2a cell lysates, human hippocampi extracts, and hippocampus and cortex extracts from mice were placed on ice and homogenized in extraction buffer (10 mM Tris–HCl pH 7.8, 0.5 mM dithiothreitol, 5 mM ATP, 0.03% Triton X-100, and 5 mM MgCl2). The lysates were centrifuged at 13.000 × g at 4 °C for 20 min. The resulting supernatants were placed on ice and assayed for protein concentrations by Bicinchoninic acid (BCA) (Bio-Rad). To determine proteasome activity, we followed the procedure previously described [30]. Briefly, extracts were adjusted to 0.5 mg/ml total protein by dilution with extraction buffer. Chymotrypsin-like activity was determined using the substrate Suc-LLVY-aminomethylcoumarin (AMC) (Sigma; 100 μM), trypsin-like activity was determined using the substrate Boc-LSTR-AMC (Sigma; 100 μM), and postglutamyl-like activity was determined using the substrate Z-LLE-β-2-naphtylamine (NAP) (Sigma; 200 μM). Assay mixtures containing 6 μg of protein substrate, and assay buffer (100 mM HEPES–KOH pH 7.5, 5 mM MgCl2, 1 mM ATP, 10 mM EGTA) which are made up in a final volume of 150 μl. For kinetic studies, incubations were performed at 37 °C for 60 min. The cleavage products AMC and NAP were analyzed, after stopping the reaction with 1 ml of 1% SDS, in a fluorimeter (excitation/emission: 333/410 nm for NAP and 380/435 nm for AMC). Background activity (caused by non-proteasomal degradation) was determined by addition of the proteasome inhibitor MG132 at a final concentration of 10 μM.

### Immunoaffinity purification of 20S proteasome under native conditions

Assembled 20S proteasome complexes were purified from N2a cells, treated or not with 200 µM of BzATP, by native IP with rabbit polyclonal MCP- antibody [32, 33] directed against the 20 S proteasome core. In each experiment, 2 × flask per treatment were used. The MCP-antibody was coupled to protein-A sepharose beads (GE-healthcare) O.N at 4C under rotatory agitation and washed five times with the BIP buffer (50 mM Tri-HCl pH 7.5, 150 mM NaCl, 0.5% NP40, 0.5 mM PMSF, 10 µg/ml Leupeptin, 10 µg/ml Pepstatin) after coupling. 24 h after treatment, cells were harvested on ice and washed by resuspension 4 times with ice-cold PBS. The pellet was then lysed in 500 µl of BIP buffer and centrifugated for 30 min at 14,000 rpm at 4 °C to remove cellular debris, protein amount was measured, and equal amounts of the supernatant were split into the tubes for the IPs. Each aliquot was incubated for 3 h at room temperature with rotary shaking with the MCP-8017.3-Sepharose antibody. The Sepharose beads were then washed with 500 µl of ice-cold BIP buffer for four times and immunoprecipitated proteins were eluted in loading buffer (SDS-LB N-ethylmaleimide to a final concentration of 25 mM). The immunoaffinity-purified protein complexes were characterized by Western blotting.

### Western blot analysis

N2a cells were lysed and homogenized for 1 h at 4 °C in lysis buffer containing 50 mM Tris/HCl, 150 mM NaCl (all salts from Sigma-Aldrich), 1% Nonidet P40 and Complete™ Protease Inhibitor Cocktail Tablets (Roche Diagnostics GmbH), pH 7.4.

For mouse samples, hippocampal protein extracts were prepared by homogenizing fresh dissected mouse hippocampi in ice-cold extraction buffer containing 20 mM Hepes, 100 mM NaCl, 50 mM NaF, 5 mM EDTA, 5 mM Na_3_VO_4_ (all salts from Sigma-Aldrich), 1% Triton X-100, okadaic acid (Calbiochem), and Complete TM Protease Inhibitor Cocktail Tablets (Roche Diagnostics GmbH), pH 7.4.

For human samples, hippocampi were obtained at the time of the autopsy, and they were immediately frozen in dry ice and stored at − 80 °C for biochemical studies. Control and AD brain samples were processed following the same protocol as described for mouse samples.

Protein content was determined by Bradford assay and 20 μg of hippocampal protein extract was electrophoresed on 10% Tris–Glycine-SDS gels and transferred to nitrocellulose membranes (Amersham Biosciences). The experiments were performed using the following primary antibodies: chicken anti-GFP (1:1000), rabbit anti-GFP (1:1000), rabbit anti-P2X7R (1:1000), rabbit anti-20S CORE (1:1000), mouse anti-β1 (1:1000), mouse anti-β2 (1:1000), rabbit anti-β5 (1:1000), mouse anti-β1i (LMP2) (1:1000), rabbit anti-β2i (MECL-1) (1:1000), rabbit anti-β5i (LMP7) (1:1000), rabbit anti-Rpt3 (1:1000), mouse anti-Rpn12 (1:1000), mouse anti-poly-ubiquinated proteins (FK2) (1:2000), rabbit anti cleaved caspase-3 (1:500), rabbit anti-NeuN (1:500), rabbit anti-phospho-GSK-3 (pS21/9) (1:1000), mouse anti-GSK3 α/β (1:1000), rabbit anti-phospho Akt (1:1000), mouse anti-Akt-total(1:1000), mouse anti-STAT3 total (1:1000), rabbit Nrf2 (1:1000), mouse anti-α-tubulin (1:10,000), mouse anti-GAPDH (1:10.000), mouse anti-β-actin (1:10,000). The membranes were washed for 10 min with PBS-Tween three times and incubated with secondary antibodies for 1 h at room temperature. Secondary antibodies used were goat anti-mouse (1:1000; 1:10,000) or goat anti-rabbit (1:1000) to horseradish peroxidase (HRP, Amersham GE Healthcare) followed by enhanced chemo luminescence detection (PerkinElmer, Waltham, MA, USA). Gel band images were captured with ImageQuant LAS 500 (GE Healthcare Life Sciences) and analyzed using ImageJ software (v1.52n, NIH, Bethesda, MD, United States). Proteins bands were also detected using the LI-COR Odyssey Classic and associated Image Pro analysis software (LI-COR Biosciences, Cambridge, UK) when membranes were incubated with fluorescent-tagged secondary antibody 700 or 800 (1:10,000) (red or green channel) following the manufacturer’s recommendation. In the figures, the representative Western blot images show only the quantified bands.

### Tissue processing for immunohistochemistry and immunofluorescence

#### Mouse brain

Mice were sacrificed by cervical dislocation and their forebrain removed. Brains were fixed in 4% paraformaldehyde and cryoprotected in 30% sucrose solution. The samples were embedded in OCT compound (Sakura) and frozen using dry ice. Finally, 30 µm floating sections were cut in coronal planes with a cryostat (CM1950, Leica Microsystems) and stored in a solution of 30% ethylene glycol, 30% glycerol, and 0.1 M PBS at − 20 °C until processed.

#### Human brain

Whole brain from control and AD subjects was separate into two hemispheres, through a sagittal interhemispheric incision, and fixed in 10% buffered formalin for at least 3 weeks. Samples (2-mm-thick) of the hippocampus were then obtained through dissection, embedded in paraffin following standard protocols (Leica, HistoCore Pearl), and later sectioned (5 μm) by using a microtome (Microm HM 355 S) and stored at − 80 °C until use.

### Immunohistochemistry and immunofluorescence

For immunohistochemical analysis, mouse sections were pre-treated for 45 min with 1% H_2_O_2_ in PBS to inactivate endogenous peroxidase. Afterwards, sections were washed in PBS, blocked for 1 h in block solution (1% bovine serum albumin (BSA), Sigma-Aldrich, 5% fetal bovine serum (FBS), and 0.2% TritonX-100, Sigma-Aldrich, in PBS) and finally incubated ON at 4 °C with chicken anti-GFP (1:500). Later, sections were incubated with avidin–biotin complex using the Elite Vectastain kit (Vector Laboratories, Burlingame, CA, USA) and chromogen reactions were performed with diaminobenzidine (DAB; Sigma-Aldrich) and 0.003% H_2_O_2_ for 10 min.

For immunofluorescence studies, mouse slices were washed in PBS, blocked for 1 h at room temperature (RT) with blocking solution, and then incubated at 37 °C for 1 h or overnight at 4 °C with primary antibodies diluted as follows: rabbit anti-P2X7 (1:100), chicken anti-GFP (1:500), mouse anti-GFAP (1:200), rabbit anti-Iba-1 (1:100), mouse anti-NeuN (1:100), rabbit β5 (1:300) in blocking solution. Subsequently, brain sections were washed with PBS buffer and incubated with secondary antibodies at the following dilutions: anti-chicken IgG labeled with Alexa 488 (1:400), anti-rabbit IgG labeled with Alexa 555 (1:400), anti-rabbit IgG labeled with Alexa 594 (1:400), anti-mouse IgG labeled with Alexa 555 (1:400) and 4′,6-diamidino-2-phenylindole (DAPI) staining (1:1000). Finally, brain sections were washed with PBS and mounted in FluorSave (Calbiochem).

For immunofluorescence studies, human sections were pre-incubated at 55 °C ON and then sequentially washed for 10 min in Xylene (catalog 131,769.1611, Panreac), ethanol 100%, ethanol 96%, ethanol 70%, and finally distilled H_2_O in order to remove paraffin and rehydrated them. Later, the same protocols described for mouse samples were followed.

### Image acquisition

Confocal images were acquired at RT with a TCS SPE microscope from Leica Microsystems equipped with a Plan Fluor 10 × dry objective lens NA = 0.30, 40 × Apochromat NA = 1.15 oil objective lens, and 63 × Apochromat NA = 1.3 oil objective lens (Leica Microsystems GmbH) and 4 different lasers lines (405, 488, 565, and 647 nm). Pictures were acquired using the Leica software LAS AF v2.2.1 software (Leica Microsystems GmbH) and representative slices were converted to TIFF files using ImageJ software. Transmitted light images were acquired using a microscope (DM 1000, Leica) with a DFC450 CCD camera (Leica Microsystems GmbH) using Leica Application Suite (v4.1). Sections were photographed with Plan 4 × dry objective lens (NA = 0.1) and insets with Plan S-Fluor 20 × or 40 × dry objective lens (NA = 0.90, Nikon) at RT.

### Cell counting

To quantify the number of total hippocampal GFP-positive cells from UbGFP mice vehicle- or BzATP-treated via i.c.v., eight-bit confocal images of both ipsilateral and contralateral hippocampus from coronal brain slices were acquired. To identify GFP-positive neurons, glia, or microglial cells, coronal sections with specific antibodies for neurons (NeuN), glia (GFAP), and microglial (Iba-1) were co-stained, and eight-bit confocal images were acquired for each marker (at least three slices per mouse and five mice per treatment). Positive cells were identified by immunofluorescence techniques and considered only those with DAPI nuclear-positive staining. Positive stained cells were considered those with a mean intensity value was > 170 on a 0–255 scale, where 0 = white and 255 = black. The cut-off value of 170 was determined from visual analysis of immunolabeling and by comparison with control (maximal level obtained with preabsorbed antibodies).

For the counting of a hippocampal cell bearing polyubiquinated aggregated in vehicle- or GSK-treated P301S mice (6–10 slices per mouse and at least 4 mice per treatment) or GFP positive cells in UbGFP or UbGFP;P301S mice (3 slices per mouse and 3 mice per genotype) parasagittal brain sections were employed. Cells bearing polyubiquinated aggregated and GFP-positive cells were identified by immunohistochemistry techniques, counting the number of them present in the whole hippocampus or in CA3 area of each slice respectively.

To quantify the number of hippocampal positive NeuN neurons, confocal images of the hippocampal CA3 area from WT, P301S, GSK-treated P301S, and P301S;P2X7^−/−^ mice were acquired from 4 different sagittal brain slices per mouse on at least 3 mice per condition. Eight-bit confocal images of two single focal planes per section were acquired. Positive stained cells were considered those with a mean intensity value was > 150 on a 0–255 scale, where 0 = white and 255 = black.

### Statistics

Data are shown as mean values ± SEM. The numbers of mice per group used in each experiment are annotated as “n” in the corresponding figure legends. Figures and statistical analyses were generated using GraphPad Prism (v8.00, www.graphpad.com). To assess whether the data met the normal distribution, the Shapiro–Wilk or Kolmogorov–Smirnov tests were used. For two-group comparison, data were analyzed with a two-tailed unpaired Student’s *t* test. For multiple comparisons, data were analyzed using a one-way ANOVA followed by Dunnett's post hoc tests when all groups were compared against a control group or one-way ANOVA followed by Tukey’s post hoc tests when all groups were compared among them. When required, a two-way ANOVA followed by Tukey’s post hoc tests was used. The statistical test used, and *p*-values are indicated in each figure legend. Significance was considered at *P ≤ 0.05, **P ≤ 0.01, ***P ≤ 0.001 or ****P ≤ 0.0001 throughout the study.

## Results

### The P2X7R modulates UPS activity by regulating the expression of 20S proteasome catalytic subunits

**Fig. 1:**
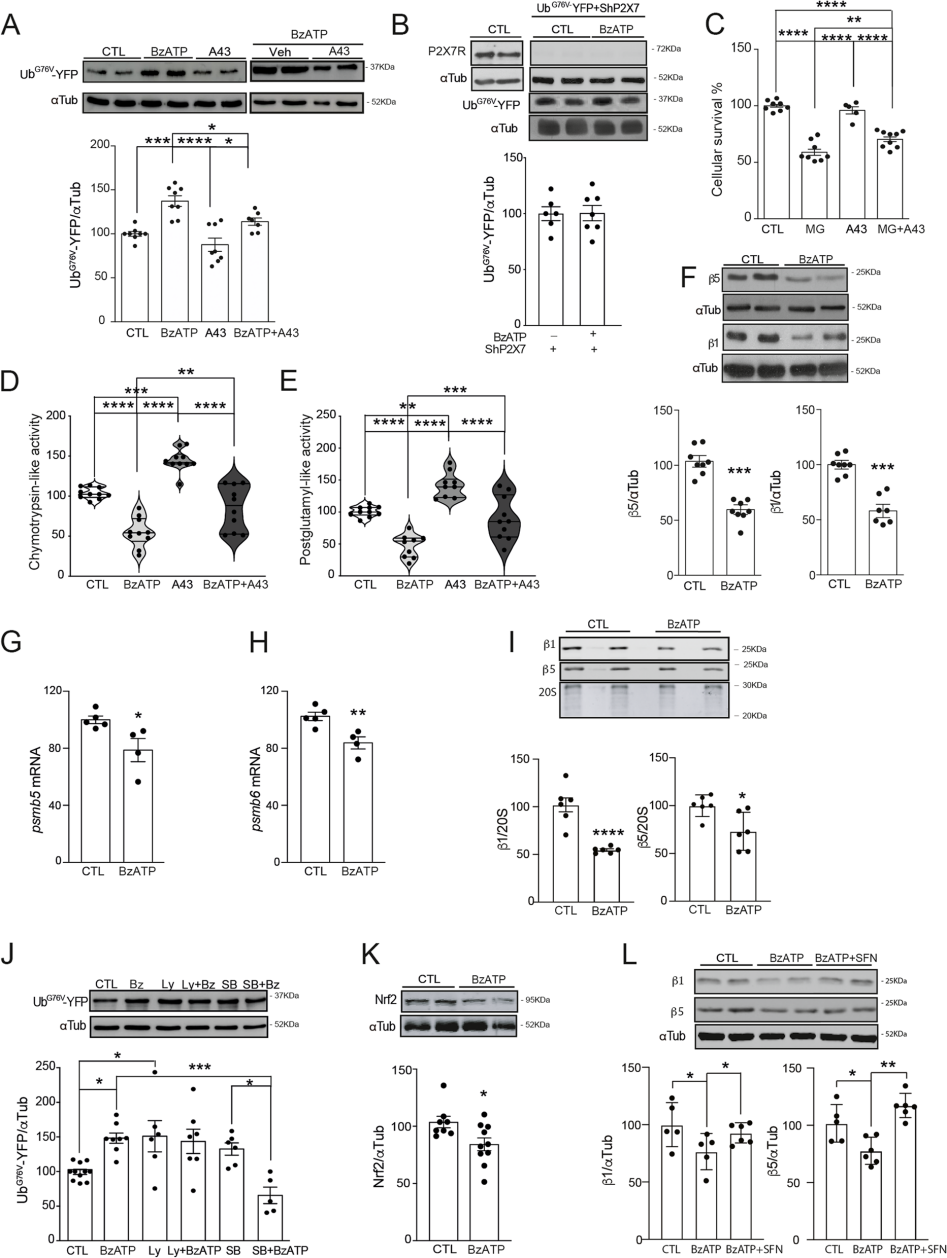
P2X7R activation in N2a cells increases proteasomal chymotrypsin-like and post-glutamyl-like activities by increasing the proteasomal subunits β5 and β1 translation and transcription and promoting its later assembly to 20S proteasomal core via IP3K/GSK3/Nfr2. **A** Representative Western blot and quantification of UPS reporter levels in Ub^G76V^-YFP transfected N2a cells stimulated with 200µM BzATP or vehicle solution (CTL), in the presence or absence of selective P2X7R antagonist 40µM A438079 (*n* = 4 in triplicated). **B** Genetic knockdown of P2X7 avoids BzATP-induced Ub^G76V^-YFP accumulation in N2a cells (*n* ≥ 3 in duplicated). Sample loading was normalized with anti-α-tubulin (α-Tub) antibody. Graphs show the quantification of Ub^G76V^-YFP levels. 100% corresponds to the Ub^G76V^-YFP protein expression detected in Ub^G76V^-YFP transfected cells (**A**) or cells co-transfected with Ub^G76V^-YFP plus shRNA P2X7 (**B**) treated with vehicle solution. **C** Percentage of N2a cells survival treated with 10 µM MG132 for 24 h in the presence or absence of 40 µM A438079 (*n* = 4 in duplicated). Measurement of chymotrypsin-like (**D**) and postglutamyl (**E**) peptidase activities assayed in N2a cells stimulated with 200µM BzATP in the presence or absence of 40µM A438079 (*n* = 3 in triplicated). 100% value corresponds to the activity detected in vehicle-treated N2a cells. In all reactions, the specificity of the fluorogenic reaction was verified by the addition of the MG132 proteasome inhibitor. **p ≤ 0.01; *** *P* ≤ 0.001; **** *P* ≤ 0.0001 using a one-way ANOVA followed by Tukey’s post hoc test. **F** Western blot analysis of β1 and β5 proteasome subunits expression in N2a cells stimulated with 200µM BzATP or vehicle solution. Quantitative PCR analysis of β5 (*psmb5*, **G**) and β1 (*psmb6*, **H**) mRNA levels in vehicle- (CTL) or BzATP-treated N2a cells. Values were normalized to endogenous GAPDH as a loading control (*n* ≥ 3 in duplicated). **I** Representative Western blot image and quantification of β5 and β1 subunits immunoprecipitated with MCP-8017.3 antibody. Protein amount was normalized to 20S core (*n* ≤ 3 in duplicated). *p ≤ 0.05; **p ≤ 0.01; *** *P* ≤ 0.001; **** *P* ≤ 0.0001; using an unpaired two tailed Student’s t-test. **J** Representative Western blot and quantification of UPS reporter levels in Ub^G76V^-YFP transfected N2a cells stimulated with 200µM BzATP in the presence or absence of selective inhibitors of intracellular kinases, the PI3K inhibitor 50 µM LY294002 (LY), or the GSK3β kinase 5 µM SB216763 (SB). Sample loading was normalized with anti-α-tubulin antibody. 100% corresponds to Ub^G76V^-YFP protein expression detected in cells treated with vehicle solution (CTL). *p ≤ 0.05; *** *P* ≤ 0.001; using a two-way ANOVA followed by Tukey’s post hoc test. **K** Representative Western blot image and quantification of Nfr2 transcription factor levels in N2a cells stimulated with 200µM BzATP or vehicle solution (*n* ≥ 4 in duplicated). *p ≤ 0.05 using an unpaired two-tailed Student’s *t*-test. **L** Western blot and quantification of β1 and β5 expression levels in N2a cells stimulated with vehicle solution (CTL) or 200 BzATP in the presence or absence of selective Nfr2 stimulator 10 µM sulforaphane. Sample loading was normalized with anti-α-tubulin antibody. 100% corresponds to β1 and β5 expression detected in cells treated with vehicle solution (CTL). Data in bar graphs represent mean ± s.e.m. *p ≤ 0.05; ** *P* ≤ 0.01; using a one-way ANOVA followed by Tukey’s post hoc test

To investigate whether P2X7Rs can regulate the UPS, we initially used two different UPS reporter proteins, a ubiquitin fusion degradation (UFD) substrate: ubiquitin G76V-yellow fluorescent protein (Ub^G76V^-YFP) [34] and the GFP protein fused to the C-terminal degradation signal CL1 (GFP-CL1) [35]. Given that both proteins are rapidly processed by the 20S proteasome, an UPS impairment should lead to an intracellular accumulation of these reporter proteins. Since previous works reported the presence of functional P2X7Rs in the N2a neuroblastoma cell line [36–38], as a first step, we decided to use this cellular model to elucidate if P2X7R may modulate the UPS system. Stimulation of Ub^G76V^-YFP transfected Neuroblastoma (N2a) cells with the P2X7R agonist, BzATP, caused a significant accumulation of the reporter protein in a time and dose-dependent way (an increase of 52.0 ± 8.4% with a pEC_50_ = 4.2 ± 0.3 M at 24 h). Consequently, we established the assay condition for in vitro studies at BzATP 200 µM for 24 h (Fig. 1A and Supplementary Fig. 1A–B). Similar results were obtained with GFP-CL1 transfected N2a cells (Supplementary Fig. 1C). The selective P2X7R antagonist A438079 avoided BzATP-induced Ub^G76V^-YFP accumulation (Fig. 1A). In agreement with these results, P2X7R selective downregulation by Sh-RNA prevented increased UPS reporter accumulation in response to BzATP (Fig. 1B). Measuring Ub^G76V^-YFP mRNA levels and transfecting N2a cells with a plasmid encoding the fusion protein Ub-M-GFP [34] (which lacks the G76V substitution so that it is rapidly cleaved to release the ubiquitin moiety and GFP) we could rule out that transcriptional or translational alterations are underlying the observed effects (Supplementary Fig. 1D–E). Further studies confirmed that the P2X7R-induced accumulation of UPS-reporter proteins was neither caused by modifications in the cellular content of 20S proteasome core or alterations in subunits composing the proteasome cap nor by decreasing cellular viability (Supplementary Fig. 1F–G). Interestingly, we found that pharmacological blockade of the P2X7R with A438072 significantly reduced the cellular death caused by MG-132-induced pharmacological blockade of the proteasome catalytic activity (Fig. 1C).

Next, we tested whether P2X7R causes UPS impairment via affecting proteasome catalytic activities. BzATP-stimulated N2a cells showed a significant decrease in 20S proteasomal chymotrypsin-like (CT-L) and postglutamyl-like (P-L) activities by 49.2 ± 8.8% and 53.1 ± 9.8% respectively (Fig. 1D, E), but not trypsin-like activity (T-L) (Supplementary Fig. 1H). A438072 prevented BzATP-induced reduction of CT-L and P-L activities (Fig. 1 D and E). The reduction of proteasomal catalytic activities was linked to a significant decrease in the expression levels of 20S proteasome constitutive catalytic subunits β5 and β1 (43.8 ± 6.8% for β5 and 41.9 ± 7.0% for β1, Fig. 1F), but not β2 subunit nor the inducible subunits β1i, β5i, β2i (Supplementary Fig. 1I). Reduction in β5 and β1 protein levels were associated with a decrease in messenger RNA levels of *psmb5* and *pmsb6* (21.2 ± 7.7% for *psmb5* and 18.6 ± 5.0% for *pmsb6*, Fig. 1G, H), without affecting *pmsb7* levels (Supplementary Fig. 1 J). Immunoprecipitation assays confirmed that P2X7R activation reduced the number of β5 and β1 catalytic subunits assembled to 20S proteasome core particle (26.8 ± 9.5% and 46.4 ± 0.8% respectively, Fig. 1I). Accordingly, overexpression of β5 or β1 subunit avoided BzATP-induced UPS reporter accumulation (Supplementary Fig. 1 K). Altogether, these results suggest that P2X7R activation causes an UPS impairment by decreasing the expression of the catalytic subunits β5 and β1 and, therefore, their assembly into the 20S proteasome core.

### The P2X7R induces UPS impairment in neurons and microglia by downregulating β5 expression via the AKT/GSK3 pathway

**Fig. 2:**
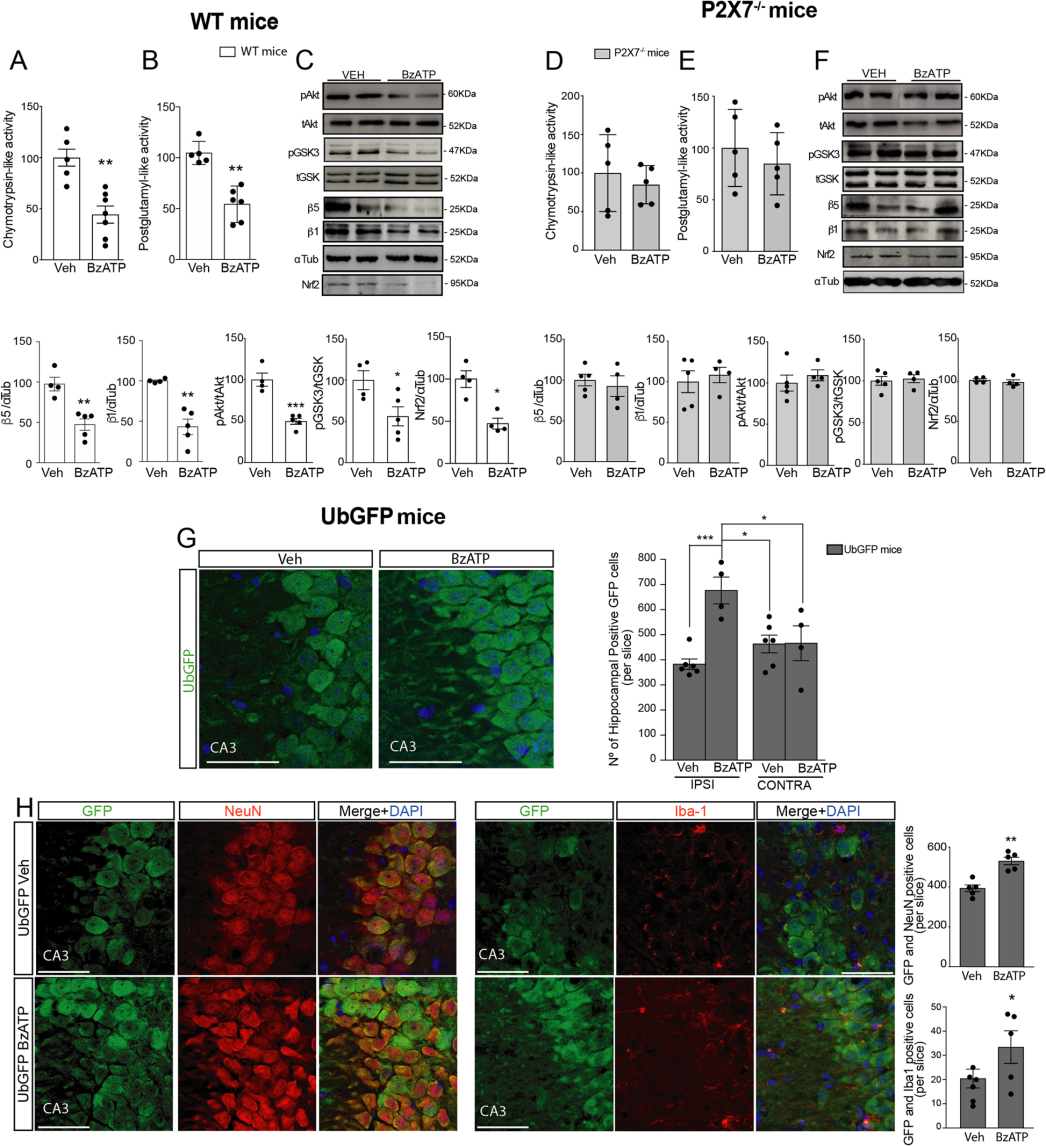
In vivo P2X7R activation decreases proteasomal chymotrypsin-like and post-glutamyl-like activities in neurons and microglia cells by reducing the proteasomal subunits β5 and β1 translation and transcription via Akt/GSK3/Nfr2. Chymotrypsin-like (**A** and **D**) and postglutamyl (**B** and **E**) activities were measured in hippocampal extracts from WT mice (**A** and **B**, white bars) or P2X7R^−/−^ mice (**D** and **E**, gray bars) treated with a single injection of 2µL 30 mM BzATP or vehicle solution (Veh) via i.c.v. for 24 h (n ≥ 4 in triplicated). 100% value corresponds to activity detected in vehicle-treated mice. In all reactions, the specificity of the fluorogenic reaction was verified by the addition of the proteasome inhibitor MG132. Representative images of Western blot using hippocampal samples from WT mice (**C**) or P2X7R^−/−^ mice (**F**) treated as above indicated and stained with antibodies anti-β5 and anti-β1 subunits, anti-phosphorylated (pAkt) or anti-total Akt, anti-phosphorylated (pGSK3) or anti-total GSK3, anti-Nfr2, and anti -tubulin. Graphs show the quantification of proteasome catalytic subunits β5 and β1, as well the levels of p-Akt, p-GSK3, and Nfr2 in WT mice (left side, white bars) and P2X7^−/−^ (right side, gray bars) mice. Membranes are probed with an anti--tubulin antibody to correct for any possible deviation on protein loading. 100% value corresponds to protein levels detected in vehicle-treated WT or P2X7^−/−^ mice respectively. Data in bar graphs represent mean ± s.e.m. *p ≤ 0.05; **p ≤ 0.01; *** *P* ≤ 0.001; using an unpaired two-tailed Student’s *t*-test. **G** Representative images of hippocampal CA3 area from UbGFP mice 24 h after being treated with a single injection via i.c.v. of 2µL 30 mM BzATP or vehicle solution and stained with antibody against GFP (green channel) and nuclear marker DAPI (blue channel). Original scale bars, 100µm. Quantification of hippocampal CA3 green cells per slice (*n* ≥ 5 mice per treatment and *n* ≥ 3 sections per mouse). *p ≤ 0.05; *** *P* ≤ 0.001; using a two-way ANOVA followed by Tukey’s post hoc test. **H** Hippocampal sections from BzATP- or vehicle-treated UbGFP mice stained with a specific neuronal marker (NeuN, red channel) and GFP (green channel) on the left side and microglial marker (Iba-1, red channel) and GFP (green channel) on the right side. Merged images showing the DAPI channel in blue are also shown. Scale bar: 100 μm. Quantification of the GFP-positive cells identified as neurons (upper) or microglial cells (down) per hippocampal slice in BzATP- or vehicle-treated UbGFP mice (*n* ≥ 5 mice per treatment and n ≥ 3 sections per mouse). Data in bar graphs depict means ± s.e.m. *p ≤ 0.05; **p ≤ 0.01; using an unpaired two-tailed Student’s *t*-test

Since previous studies reported that P2X7R is involved in the regulation of the phosphatidylinositol 3-kinases (PI3K)/Akt/GSK3 axis [31, 36], we wanted to explore whether this pathway plays a role in P2X7R-induced UPS regulation. To this end, Ub^G76V^-YFP transfected N2a cells were treated with a selective antagonist of the GSK-3β kinase, 5 μM SB216763 (SB), or the selective PI3K inhibitor, 50 μM LY294002 (LY), for 20 min before their stimulation with BzATP or vehicle solution. Of note, LY-induced a similar accumulation of the UPS reporter when compared to BzATP alone; no additive effect was, however, detected when both compounds were assayed together (Fig. 1J). As for SB, no significant accumulation of UPS reporter was detected when assayed alone, SB treatment prevented, however, BzATP-induced UPS impairment (Fig. 1J). In line with these results, BzATP reduced the phosphorylation of GSK3β in Ser 9/21 residues, which has been described to decrease kinase activity. Additionally, phosphorylation of the GSK3 upstream kinase Akt (Supplementary Fig. 2A) was also decreased. Finally, based on previous studies reporting that *psmb5* may be transcriptionally regulated by the GSK3-sensitive transcription factors; signal transducer, and activator of transcription 3 (STAT3) [39] or Nfr2 [40], we analyzed the expression levels of both transcription factors in our experimental conditions. P2X7R activation reduced the cytosolic Nrf2 levels, which was not observed for STAT3 levels N2a cells treated with BzATP (Fig. 1K and Supplementary Fig. 2B). Supporting these data, later studies using sulforaphane, an activator of Nrf2 [41], confirmed that the pharmacological activation of Nrf2 avoids the BzATP-induced decrease of the expression of the β5 and β1 subunits (Fig. 1L). Taken together, our results indicate that P2X7R activation reduces UPS activity by decreasing the expression of proteasome β5 and β1 subunits by reducing cytosolic Nrf2 levels via PI3K/Akt/GSK3 pathway. Next, we wondered how P2X7R-induced UPS regulation impacts on brain physiology in vivo. Here, we focused on the hippocampus where P2X7R expression has been widely described [42]. WT and P2X7R knockout mice (P2X7R^−/−^) were intracerebroventricular (i.c.v.) injected with BzATP at 1.43 mg/Kg of body weight or the same volume of vehicle solution (Veh). Twenty-four hours after treatment, we observed that BzATP administration induced a significant reduction in hippocampal CT-L and P-L activities (a decrease of 55.7 ± 11.8% and 44.4 ± 11.4% respectively compared to Veh-treated mice, Fig. 2A, B) and in β5 and β1 levels in WT mice (53.2 ± 12.3% and 56.7 ± 12.8% less β5 and β1 expression compared to Veh-treated mice, Fig. 2C). Treatment with BzATP did not affect cellular viability (Supplementary Fig. 3D). In good agreement with our in vitro studies, a reduction in hippocampal *psmb5* and *psmb6,* but not in *psmb7* mRNA levels, were detected in BzATP-treated WT mice (Supplementary Fig. 3A–C). BzATP also significantly reduced the phosphorylation of hippocampal GSK3β and Akt kinases (43.8 ± 16.5% for GSK3β and 50.3 ± 8.0% for Akt) and Nrf2 expression levels (52.7 ± 14.1%) (Fig. 2C). None of these alterations were detected in BzATP-treated P2X7R^−/−^ mice (Fig. 2D–F), confirming their P2X7R-dependency. P2X7R^−/−^ mice also showed no significant basal changes in any of the proteasomal activities tested (Supplementary Fig. 3E–G). In summary, our in vivo data support our in vitro data suggesting that the P2X7R regulates the activity of the UPS in the brain in vivo by regulating the transcription of β5 and β1 catalytic subunits via the Akt/GSK3/Nrf2 pathway.

To identify which cellular lineages are sensitive to P2X7R-mediated UPS regulation, we resort to using UbGFP mice, which ubiquitously express the UPS reporter Ub^G76V^-GFP. Twenty-four hours after UbGFP mice were i.c.v. injected with BzATP or vehicle solution, the mice were sacrificed and analyzed. As expected, BzATP-treated UbGFP mice showed a significant increase in the accumulation of UPS reporter protein in hippocampal cells when compared to Veh-treated UbGFP mice. Of note, this accumulation was only observed in the ipsilateral hemisphere but not in the contralateral one (Fig. 2G). Double immunolabeling of hippocampal brain slices using specific markers for astrocytes (GFAP), microglia (Iba-1), and neurons (NeuN) showed that BzATP-mediated P2X7R activation led to the accumulation of the Ub^G76V^-GFP reporter in both neurons and microglial cells (Fig. 2H), but not in astrocytes (Supplementary Fig. 3H).

### P2X7R inhibition reverts UPS impairment associated with Alzheimer’s disease

**Fig. 3:**
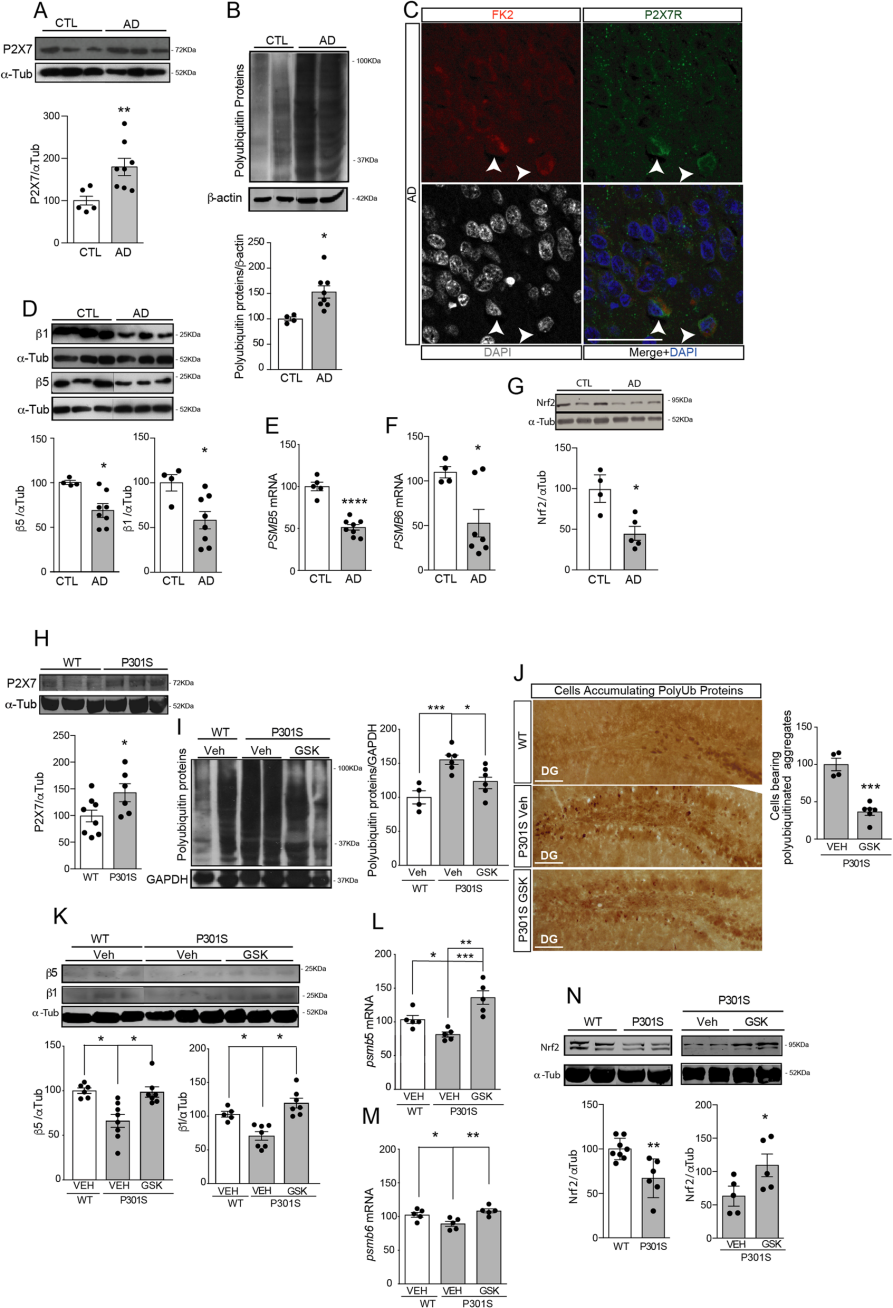
In vivo P2X7R inhibition reverts the UPS dysfunction that P301S mice mimic from AD patients. **A** Representative Western blot and quantification of P2X7R (**A**) and polyubiquitin proteins (**B**) using homogenates from hippocampal necropsies from Alzheimer’s disease (AD) patients and non-affected individuals (CTL). Graphs show the quantification of P2X7R (**A**) or polyubiquitin (**B**) protein expression in hippocampal homogenates from AD (*n* = 8) and control (*n* = 5). α-tubulin (α-Tub) or β-actine expression was used as loading control for normalization purposes. 100% value corresponds to the averaged amount of P2X7 or polyubiquitinated proteins (polyUb) detected in CTL. **C** Representative hippocampal sections from AD patients stained with polyUb marker (FK2, red channel), P2X7R (green channel), and DAPI (gray channel). Merged images are also shown. Arrowheads indicate neural cells expressing high levels both of P2X7R and polyUb. Scale bar: 50 μm. **D** Western blot and quantification of β5 and β1 protein levels in homogenates from hippocampal AD patients (*n* = 8) and control individuals (*n* = 4). Quantification of β5 mRNA (*PSMB5*) (**E**) and β1 mRNA (*PSMB6*) (**F**) in the hippocampus from human controls (*n* = 4) and AD patients (*n* ≥ 7). In all cases, the 100% value corresponds to the averaged amount of protein or messenger of β5 and β1 detected in the hippocampi of non-affected individuals. **G** Representative Western blot image and quantification of Nfr2 transcription factor both in human controls (*n* = 3) and AD patients (*n* = 5). Data in bar graphs represent mean ± s.e.m. *p ≤ 0.05; **p ≤ 0.01; **** *P* ≤ 0.0001 using an unpaired two-tailed Student’s *t*-test. **H** Representative immunoblot of P2X7R protein levels in hippocampal homogenates from 9-month-old P301S and wild-type (WT) mice. -tubulin (α-Tub) was used as a loading control. Graph shows P2X7R protein levels in P301S and WT mice (*n* = 5 mice per genotype). **I** Western blot and quantification of polyubiquitinated proteins using hippocampal homogenates from 9-month-old wild-type (WT) mice and P301S mice treated with Vehicle (Veh) or GSK 1482160A (GSK) for 3 weeks. GAPDH was used as a loading control. 100% value corresponds to the averaged amount of polyubiquitinated proteins (polyUb) detected in Veh-WT mice (*n* = 4–6 per genotype and condition). **J** Representative images of hippocampal slices stained with FK2 antibody labeling polyubiquitinated proteins in Veh- or GSK-treated P301S mice. Scale bar: 100 μm. Graph shows the number of hippocampal cells bearing intracellularly polyubiquitinated aggreges in Veh- or GSK-treated P301S mice (*n* = 4–6 per genotype). *** *P* ≤ 0.001 using an unpaired two-tailed Student’s *t*-test. **K**) Western blot and quantification of β5 and β1 protein levels in hippocampal homogenates from Veh-WT mice, Veh-P301S, and GSK-P301S mice (≥ 5 mice per treatment and genotype). Quantification of β5 mRNA (*psmb5*) (**L**) and β1 mRNA (*psmb6*) (**M**) in the hippocampus from Veh-WT mice, Veh-P301S and GSK-P301S (*n* ≥ 5 per genotype and treatment). In all cases, the 100% value corresponds to the averaged amount of protein or messenger of β5 and β1 detected in the hippocampi of Veh-WT mice. *p ≤ 0.05; **p ≤ 0.01; *** *P* ≤ 0.001 using a one-way ANOVA followed by Tukey’s post hoc test. **N** Representative Western blot image and quantification of Nfr2 transcription factor in the hippocampus from Veh-WT mice, Veh-P301S, and GSK-P301S (*n* ≥ 4 per genotype and treatment). 100% value corresponds to the average amount of Nrf2 detected in the hippocampi of Veh-WT or GSK-P301S mice. Data in bar graphs represent mean ± s.e.m. *p ≤ 0.05; **p ≤ 0.01; using an unpaired two-tailed Student’s *t*-test

Since UPS impairment has been recognized as the hallmark of a wide range of neurodegenerative disorders, including AD [14, 43], a disease in which patients show higher P2X7R expression levels [24, 25, 27], we wonder whether increased P2X7R signaling may contribute to the AD-associated UPS impairment. Our analysis confirmed that AD patients presented an increased P2X7R expression (79.6 ± 25.3%) and a higher amount of polyubiquitinated proteins (53.1 ± 17.7%) in AD patients when compared to age-matched non-AD-affected individuals (CTL) (Fig. 3A, B). Interestingly, using immunofluorescence analyses we observed that those hippocampal cells expressing high P2X7R levels achieve higher levels of polyubiquitylated proteins than their neighboring cells (Fig. 3C). Additional studies revealed that AD patients showed lower CT-L activity (25.8 ± 7.9% less), and β5 protein expression levels (32.2 ± 7.7% less) and reduced *PMSB5* mRNA levels (48.7 ± 5.9% less) than CTL (Fig. 3D, E and Supplementary Fig. 4A). Of note, although AD patients showed a significant reduction of β1 levels in both protein and messenger RNA, only a slight reduction in P-L was detected (Fig. 3D and F and Supplementary Fig. 4B). AD patients did not show significant changes in the inducible subunits β5i and β1i (Supplementary Fig. 4C, D). Finally, AD patients showed lower hippocampal Nrf2 levels than CTL (Fig. 3G).

To evaluate the relevance of P2X7R in UPS dysfunction associated to AD, we treated P301S mice with the selective P2X7R antagonist GSK 1482160A (GSK, 100 mg/kg, i.p) or the same volume of vehicle solution (Veh) for 3 weeks. Treatments were started at the age of 9 months, when P301S mice display increased P2X7R expression in the brain (Fig. 3H), accumulation of polyubiquitinated proteins (Fig. 3I), and an increased number of cells containing ubiquitinated aggregates in the hippocampus (Fig. 3J), similar to what has been observed in human AD patients. P301S mice also showed reduced CT-L and P-L proteasomal activities compared to WT mice (58.4 ± 17.6% and 20.5 ± 7.5 less respectively), lower protein expression levels of β5 and β1 subunits (a 33.8 ± 8.9% and 32.2 ± 8.3%, less respectively), reduced mRNA levels of *psmb5* and *pmsb6* (a decrease of 22.2 ± 6.9% and 13,2 ± 5.1%, respectively) and lower Nrf2 levels (Fig. 3K–N and Supplementary Fig. 4E–F). Remarkably, in vivo P2X7R blockade prevented the accumulation of the polyubiquitinated proteins, reduced the number of hippocampal cells presenting FK2 intracellular staining aggregates, prevented the decrease of CT-L and P-L activities by avoiding the reduction of β5 and β1subunits by normalizing the mRNA levels of *psmb5* and *psmb6* in P301S mice and increased Nrf2 levels (Fig. 3I–N and Supplementary Fig. 4E–F).

**Fig. 4:**
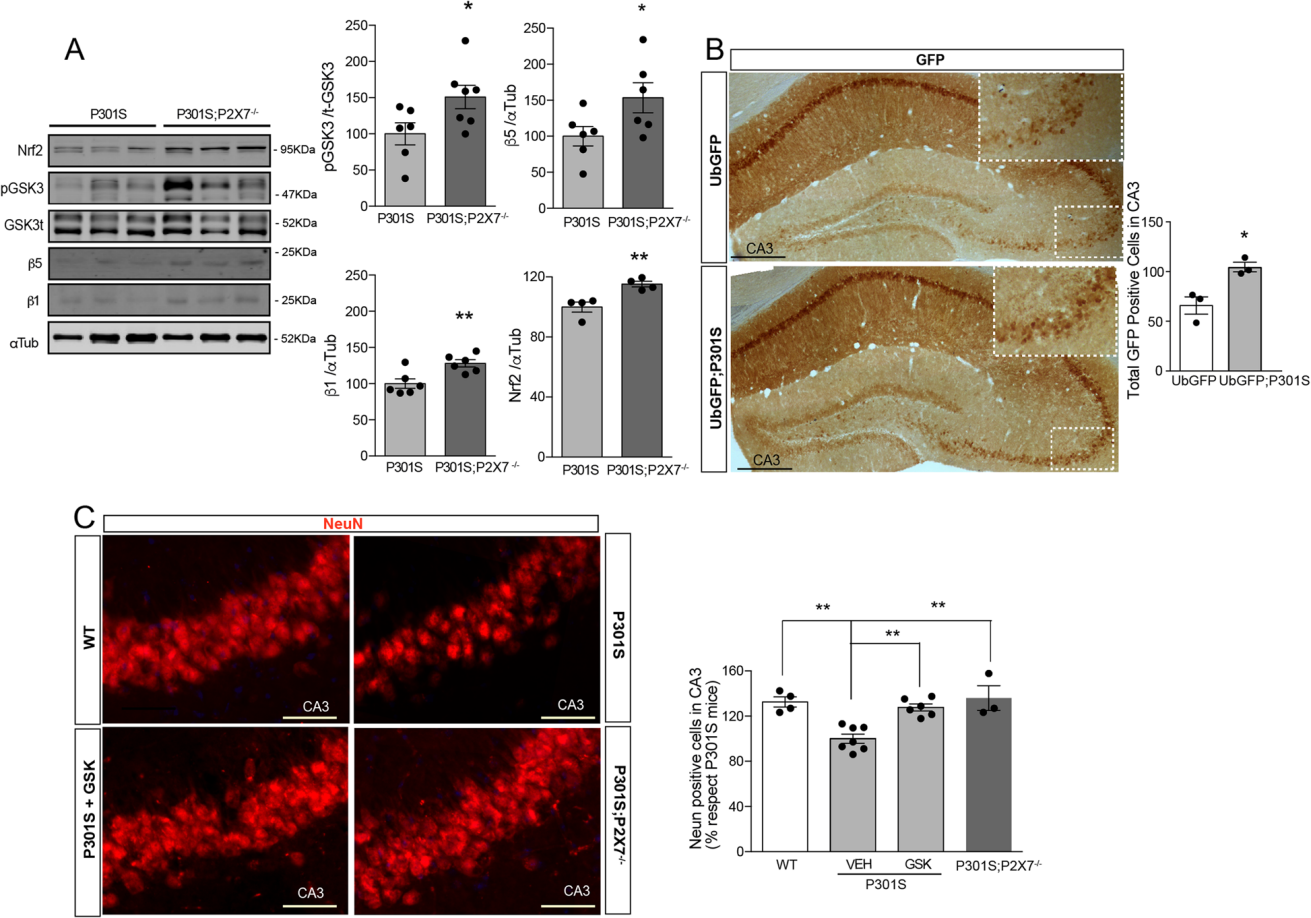
Genetic P2X7R knockdown rescues UPS dysfunction associated with the AD’s mouse model, P301S mice. **A** Representative immunoblots of Nfr2, p-GSK3, total GSK3 (tGSK3), β1, and β5 protein levels in hippocampal homogenates from P301S and P301S;P2X7^−/−^ mice (*n* = 4–7 mice per genotype). Graphs show p-GSK3β, o β5, β1, and Nfr2 protein levels using -tubulin or tGSK3β signal for normalization purposes. The 100% value corresponds to p-GSK3β, β5, β1, and Nfr2 levels detected in the hippocampus of P301S mice. **B** Representative images of hippocampal slices from UbGFP mice and UbGFP;P301S mice stained with anti-GFP antibody. Scale bar: 200 μm. Inserts show magnification X2 of hippocampal CA3 area limited by square with discontinuous sides. Graph represents the quantification of GFP positive cells in CA3 area (*n* = 3 per genotype with at least 3 slices per mice). * *P* ≤ 0.05; ** *P* ≤ 0.01; using an unpaired two-tailed Student’s *t*-test. **C** Representative immunofluorescence images of hippocampal CA3 area stained with anti-NeuN antibody from WT, P301S, GSK-treated P301S, and P301S;P2X7^−/−^ mice. Scale bars 50 μm. Graphs show the quantification of the neuronal hippocampal cell identified as NeuN positive (*n* ≥ 3 mice per genotype and treatment and *n* ≥ 4 slices per mice). Data in bar graphs represent mean ± s.e.m. *p ≤ 0.05; using a one-way ANOVA followed by Tukey’s post hoc test

To confirm the potential therapeutic role of targeting the P2X7R, we evaluated how genetic depletion of *P2X7* affects Tau-induced UPS dysfunction. To this end, P301S mice were crossbred with P2X7R knock-out mice (P2X7^−/−^) [44]. When we analyzed this newly generated transgenic line (P301S;P2X7^−/−^) we observed that the absence of P2X7Rs caused a significant rise of GSK3 phosphorylation (51.0 ± 16.2%), an increase in β5 and β1 subunits levels (53.5 ± 19.8% and 28.0 ± 4.9%, respectively), and similar levels of Nrf2 (Fig. 4A). Importantly, P301S;P2X7^−/−^ mice showed a lower number of cells with FK2-positive intracellular aggregates than P301S mice (Supplementary Fig. 4G). Altogether, our data indicate that a genetic *P2*rx7 depletion avoids Tau-induced UPS dysfunction by reduction of β1 and β5 levels because it promotes the inactivation of GSK3 kinase, stabilizing Nrf2 by inhibiting its degradation.

Finally, to identify which hippocampal area is more sensitive to an AD-associated UPS impairment, we crossbred P301S mice with UbGFP reporter mice. Comparative analysis showed that UbGFP;P301S mice have a higher number of hippocampal cells-accumulating UPS reporters than their control UbGFP littermates in the CA3 area (Fig. 4B), a location where P301S mice suffer significant neuronal loss (Fig. 4C). In agreement with a recent report [25] we found that in vivo pharmacological o genetic P2X7R blockade promotes a significant neuronal survival in hippocampal CA3 area of P301S mice (Fig. 4C). Together, these results suggest that P2X7R is involved in the UPS impairment associated with AD and that its blockade may prevent cellular death caused by an UPS impairment.

## Discussion

**Fig. 5:**
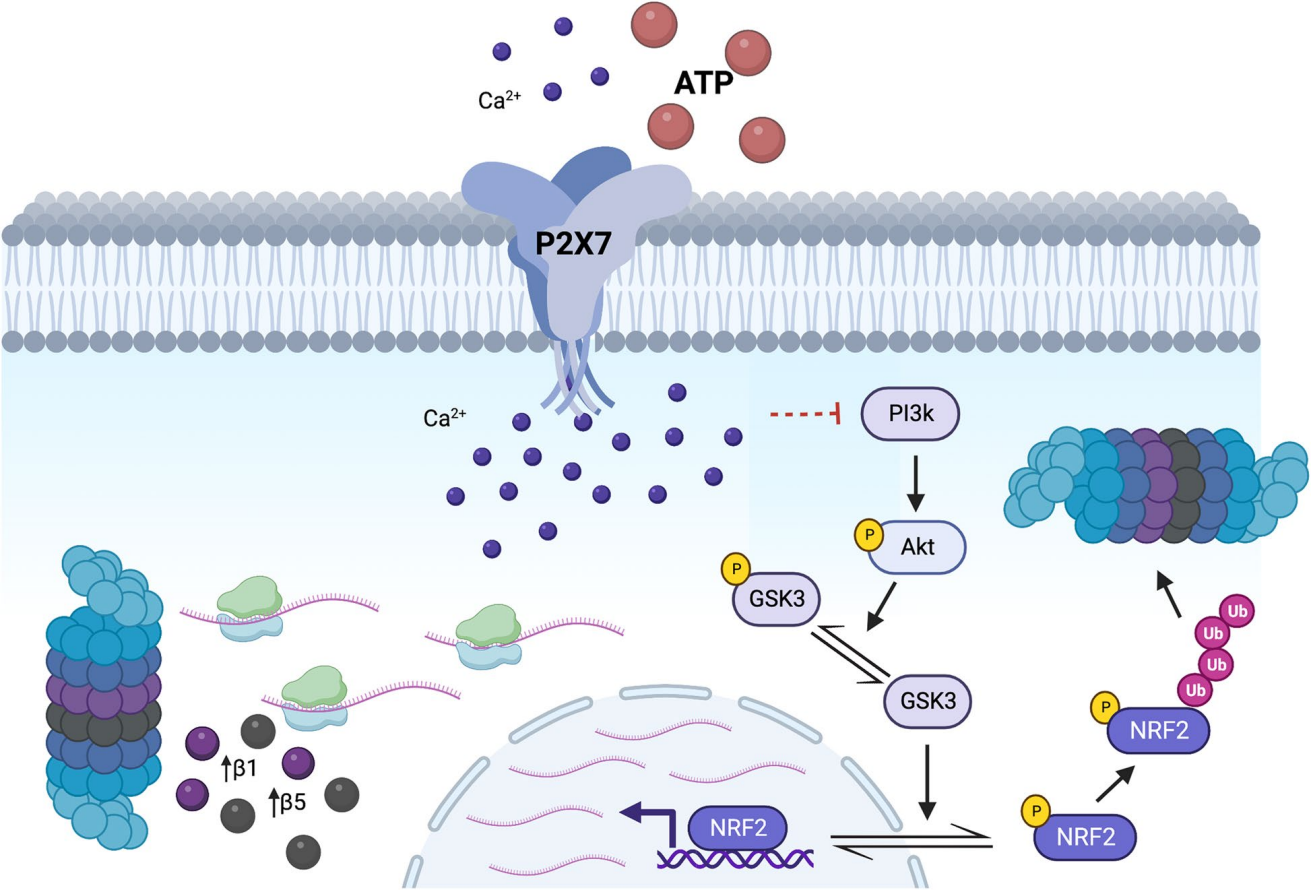
Schematic representation of the P2X7R’s signaling pathway through which it regulates UPS activity

In the present work using in vitro and in vivo approaches we report, for the first time, that extracellular nucleotides modulate the UPS via the P2X7R. This capacity is due to P2X7R activation repressing the transcription of catalytic proteasomal subunits β5 and β1 via the intracellular Akt/GSK3/Nfr2 pathway (Fig. 5). Our data also suggest P2X7R dysregulation may be underlying UPS dysfunction associated with tauopathies, specifically AD.

Mounting evidence suggests that neurodegenerative diseases, among these AD, evolve due to a failure in protein degradation systems [43]. In this regard, AD patients show alterations in protein ubiquitylation [8], diminished proteasomal activity [13, 14], and alterations in the composition of the proteasome 26S, with a reduction of the β5 subunit among others [45]. Most likely all these alterations led to the accumulation of polyubiquitinated aggregates observed in postmortem brains of AD patients [3]. However, why the proteasome fails, and which factors may contribute to that failure is still poorly understood. It is also unclear whether the AD-associated UPS dysfunction-triggered factors are intracellular or extracellular [8]. As in other neurodegenerative diseases, AD has been associated with the prolonged maintenance of a state of neuroinflammation. Here we report that one of the neuroinflammation-associated danger-associated molecules patterns (DAMPs), ATP, impacts on AD-associated UPS dysfunction. We found that in vivo P2X7R activation reproduces in WT mice, and the proteasomal dysfunction was detected both in P301S mice and in AD patients. Accordingly, P2X7R activation decreases transcription and translation of the proteasomal subunits β5 and β1, which leads to reduced CT-L and P-L activities and increases intracellular polyubiquitinated proteins in WT mice. Interestingly, previous works have reported that P2X7R is upregulated in tauopathies [24, 25, 27] and that neurodegenerative-associated neuroinflammation increases extracellular ATP levels in sufficient amounts to activate P2X7R [46]. Supporting the idea that enhanced extracellular ATP levels associated with neuroinflammation are involved in AD-related UPS impairment, we reported that in vivo P2X7R pharmacological or genetic blockage reverted proteasomal dysfunction in P301S mice. In agreement with our results, previous studies reported that neuroinflammation impacts on UPS by decreasing proteasomal function [4–6]. In this line, previous studies reported that extracellular nucleotides can modulate UPS disturbances associated with neuroinflammation. In these previous works, it has been reported that selective P2Y2R activation reversed proteasomal dysfunction associated with Lipopolysaccharide (LPS)-induced neuroinflammation in astrocytes [4, 30]. Nevertheless, our recent findings suggest that extracellular nucleotides might exert a more complex regulation on proteasomal functionality, depending on what type of purinergic receptors are expressed by the cell and the concentration that these compounds would reach in the extracellular space. For example, under pathological condition like neuroinflammation or cellular damage, nucleotides reach high concentrations at brain parenchyma, which allow them to stimulate the low-affinity P2X7R expressed by microglia cells or neurons on the affected area, decreasing the proteasomal activity in those cells. Contrarily, under non-pathological conditions, extracellular nucleotides concentration is more balanced, and they might activate higher affinity P2 receptors, such as the metabotropic P2Y2 receptor (P2Y2Rs) expressed in astrocytes, which in turn would cause neuroprotective effects by promoting the expression of proteasomal β5 subunit via the Src/ERK pathway. These antagonist effects on UPS are well matched with the opposite effects reported for P2Y2 and P2X7 in AD [38]. Hence, while P2Y2R is linked to neuroprotective effects [47], on the contrary, P2X7R is related to neurodegenerative processes [22, 23]. Accordingly to these opposite effects, it has been shown that P2Y2 haploinsufficiency increases senile plaques loading and enhances Aβ levels in the hippocampus of an amyloid mouse model [48], whereas the P2X7R pharmacological or genetic blockage reduces the number and size of amyloid plaques by reducing Aβ levels [21, 36]. In good agreement with these results reduced P2Y2 and increased P2X7R levels were found in AD patients [22, 23, 47].

It is well established that proteasomal dysregulation besides leading to aberrant protein aggregation or accumulation, ultimately may compromise cell viability and lead to cell death [49, 50]. Bolstering the regulatory role that P2X7R plays on proteasomal function, we found that raising proteasomal activity by inhibiting P2X7R, cellular death induced in N2a cells by the pharmacological inhibition of proteasome is reduced. In agreement with this neuroprotective effect, we found that in vivo pharmacological or genetic P2X7R blockage reduced the neuronal death in hippocampal CA3 area in P301S mice, as previously reported [25]. Interestingly CA3 area is where we found a significant UPS dysfunction in UbGFP;P301S mice and other groups reported that P301S mice show an important neuronal death [51]. Although these data might suggest that the observed beneficial effects on neuronal survival induced by P2X7R blockade were due to a cell-autonomous mechanism, we could not forget that P2X7R is upregulated in tauopathies, mainly in microglial cells [24, 25, 27]. Therefore, similar to what was observed in neuronal-like N2A cells, it is reasonable to think that the increased expression of P2X7R in microglial cells might be impacting the expression of catalytic subunits of the proteasome and, thus, contributing to a UPS dysfunction in this cellular lineage. Furthermore, although in the UbGFP; P301S mice, we observed that the accumulation of the UPS reporter occurs in cells with neuronal morphology, formerly it was reported that proteasome inhibition increases the neuroinflammatory response in microglial cells [52]. Consequently, it would be possible to think that P2X7R might also contribute to Tau-induced neuroinflammation by promoting the dysregulation of UPS in microglial cells. So, the in vivo observed beneficial effects may result from the dual impact that P2X7R inhibition has in both neurons and microglial cells. Supporting this hypothesis, we did not detect, at least under our experimental conditions, P2X7R activation induced-UPS dysfunction is enough to cause significant cellular death in vitro. Thus, besides the UPS dysfunction induced by P2X7R neuronal activation, other signals affecting the UPS, such as microglial secreted interleukins, would be required to reach neurons to cause enough UPS impairment to lead to cellular death. In good agreement with this hypothesis, we found that strong UPS dysfunction, for example, caused by the proteasome inhibitor MG132, is partially reverted by P2X7R blockage. Nevertheless, additional studies should be done to determine the specific contribution that P2X7R activation in neurons or microglial cells plays in Tauopathy-associated cell death.

As previously reported, we found that P2X7R activation mobilizes the intracellular PI3K/Akt/GSK3β pathway [31, 36], but here, we also observed P2X7R activation reduces the cytoplasmic levels of transcription factor Nrf2, which may regulate β5 and β1 subunits expression [53]. Since GSK3β phosphorylates Nrf2 favoring its proteasomal degradation [54], P2X7R activation might reduce Nrf2 cytosolic levels by promoting its proteasomal degradation. Supporting this hypothesis, sulforaphane-induced Nrf2 activation avoids P2X7R-induced β5 and β1 subunits reduction. Given that it was reported that a Nrf2 blockage decreases *psmb5* and β5 in a mouse model mimicking Parkinson’s neurodegenerative disease [55], it is tempting to speculate that P2X7R upregulation associated with tauopathies may be contributing to β5 and β1 downregulation observed in these diseases. Besides, considering the data reported here, we suggest that under physiological conditions, basal P2X7R activation would favor proteasomal Nrf2 degradation. However, because the reduction of Nfr2 causes a decrease in the translation of the β5 and β1 subunits, and thus, a reduction in their assembly into the proteasome, the proteasomal activity will ultimately be affected, leading to a decrease of proteasomal degradation of Nrf2, establishing in this way, a self-regulative loop. Considering this context, we hypothesize that sustained activation of upregulated P2X7R may disbalance this self-regulative loop and thus contribute to the proteasomal dysfunction observed in AD and other neurodegenerative diseases. Supporting our results other groups have reported that pharmacological Nrf2 activation improves cognitive deficits developed in both mice [56, 57], and *Caenorhabditis elegans* model of AD [58]. The mentioned improvement was linked with an increase in CT-L activity associated with an increased β5 expression [57, 58]. Similarly, it was also reported that Nrf2 activation enhances CT-L and C-L activities in a UPS reporter mouse and promotes the degradation of mutant huntingtin protein in Huntington’s disease (HD) derived cells reducing its associated cytotoxicity [41]. In agreement with our results, both HD patients and several mouse models mimicking this disease show P2X7R upregulation [59, 60], and proteasomal changes [61] in the most affected brain areas.

Over the last decades, several groups have evaluated different approaches aimed at increasing proteasome-mediated proteolysis to revert neurodegenerative disease-associated UPS dysfunction. These strategies include proteasomal activation by (i) agonist-induced conformational alteration (small molecules or peptides-based proteasome agonists); (ii) modulation of posttranslational modifications (small molecule kinase modulators or deubiquitinase inhibitors); (iii) genetic manipulation (upregulation of proteasomal activity through subunits overexpression, Nfr2 activation, or cap upregulation and generation of open-gate mutants); and (iv) other mechanisms (for review see [62]). Although the first results of these strategies seem promising, they also shed some challenges and considerations facing their future applicability. It has been proposed that these approaches may cause a disbalance of the crosstalk between UPS and autophagy [63]. Besides, there is a lack of selective proteasomal activators (the current small molecule proteasome activators have been identified by screening clinical collections of drugs, eliminating from those their activity on other targets but maintaining their activity at the proteasome[62]), and there are concerns about their possible toxicity. In this line, it has been reported that a non-selective Nfr2-mediated upregulation of proteasome activity might promote tumorigenesis [64]. All these concerns point out the necessity to develop new safer and more efficient therapeutic strategies to revert neurodegenerative disease-associated UPS dysfunction.

Recent studies carried out by us, and other groups demonstrated Tau-induced toxicity causes P2X7R upregulation in both human tauopathies patients and mouse models mimicking these pathologies [24, 25, 27]. Besides, they demonstrate pharmacological or genetic P2X7R blockade leads to i) reversion of Tau-induced behavioral alterations, ii) reduction of intracellular hyperphosphorylated Tau loading, iii) reductions of the neuronal loss in the most affected hippocampal areas, and iv) no side effects associated to this approach have been reported. Here we report, for the first time, that additionally to the above-mentioned beneficial effects, in vivo P2X7R blockade also reverts AD-associated UPS dysfunction. Furthermore, we provide new evidence about the molecular mechanisms underlying AD and other tauopathies.

## Conclusions

Our results demonstrate that (i) P2X7R negatively modulates UPS activity by downregulating the transcription of proteasomal catalytic subunits β1 and β5 through the PI3K/Akt/GSK3/Nrf2 axis, (ii) Pharmacological blockage of P2X7R reduces the cell death induced by UPS impairment, (iii) in vivo P2X7R activation induces UPS dysfunction in hippocampal neuronal and microglial cells similar to that observed in human AD patients, and (iv) pharmacological and/or genetic P2X7R blockade prevents UPS impairment in P301S mice and reduces the hippocampal neuronal death, especially in CA3, the most sensitive hippocampal area to Tau toxicity-induced UPS impairment. Therefore, our work demonstrates the sustained and aberrant activation of P2X7R caused by Tau-induced neuroinflammation contributes to the UPS dysfunction and neuronal death associated with AD, especially in the hippocampus. Moreover, we provide, for first time, solid evidence supporting extracellular nucleotide signaling via P2X7R regulates cellular proteostasis, and tau-induced toxicity compromises cell viability by deregulating this new signaling pathway.

## Supplementary Information

**Additional file 1:** **SupplementaryFigure 1.** P2X7R activationcauses a time- and dose-dependent reduction in UPS activity in N2a cells butdoes not affect the cellular translation or transcription capacities, the proteasomalcatalytic subunit β2, or the total amount of proteasome nor for affecting thecell viability. **Supplementary figure 2.** P2X7R activation induces dephosphorylation of Akt and GSK3β kinases in N2acells but does not modify the cytosolic STAT3 levels. **Supplementary figure 3.** In vivo P2X7R activation reduces the β5 andβ1 but does not β2 mRNA levels. Astrocytes are not affected by P2X7R inducedUPS regulation. **Supplementary figure 4.** Human AD patients present a reduced hippocampal chymotrypsin-like activity, butnot post-glutamyl-like activity nor inducible β5i or β1i expression levels. Invivo P2X7R blockage reverts the decreased chymotrypsin-like andpost-glutamyl-like proteasomal activities and the number of hippocampal cellsbearing polyubiquitinated aggregates in P301S mice.

**Additional file 2:** **Supplementary Table 1.** Information of Control, Alzheimer’s, and Pick’s disease cases analyzed

## Data Availability

The datasets used and/or analyzed during the current study are available from the corresponding author on reasonable request.

## Abbreviations

UPS: Ubiquitin-proteasome system
P2X7R: Purinergic P2X7 receptor
UbGFP mice: UPS reported mice
AD: Alzheimer’s disease
NFTs: Neurofibrillary tangles
GSK3: Glycogen synthase kinase 3
Nfr2: Nuclear factor erythroid 2-related factor 2
Ub^G76V^-YFP: Ubiquitin G76V-yellow fluorescent protein
GFP-CL1: GFP protein fused to the C-terminal degradation signal CL1
N2a: Neuroblastoma cell line
CT-L: Proteasomal chymotrypsin-like activity
P-L: Proteasomal postglutamyl-like activity
T-L: Proteasomal trypsin-like activity
PI3K: Phosphatidylinositol 3-kinases
LY: Selective PI3K inhibitor LY294002
SB: Selective antagonist of the GSK-3β kinase SB 216763
STAT3: Signal transducer and activator of transcription 3
i.c.v.: Intracerebroventricular
Veh: Vehicle solution
GFAP: Glial fibrillary acidic protein
Iba-1: Ionized calcium-binding adaptor molecule 1
NeuN: Neuronal nuclei
CTL: Non-AD-affected individuals
GSK: Selective P2X7R antagonist GSK 1482160A
BzATP: Selective P2X7R agonist 2′(3′)-O-(4-benzoylbenzoyl)-ATP
P2X7^−/−^: P2X7R knock-out mice
P301S;P2X7^−/−^: P301S mice, overexpresses mutated tau protein and that reproduces an AD-like Tauopathy, P2X7R deficient P301S mice
DAMPs: Neuroinflammation-associated danger-associated molecules patterns
LPS: Lipopolysaccharide
P2Y2R: Metabotropic P2Y2
HD: Huntington’s disease
GAPDH: Glyceraldehyde-3-phosphate dehydrogenase
AMC: Aminomethylcoumarin
NAP: Naphtylamine
BSA: Bovine serum albumin
FBS: Fetal bovine serum
DAB: Diaminobenzidine
DAPI: 4′,6-Diamidino-2-phenylindole
