# Supplementary material for: P2X7 receptor inhibition ameliorates ubiquitin–proteasome system dysfunction associated with Alzheimer’s disease
Source: Alzheimers Res Ther. 2023 Jun 7;15:105. doi: 10.1186/s13195-023-01258-x (PMC10245610; doi:10.1186/s13195-023-01258-x)
Supplement: Supplementary file 1 — Additional file 1: SupplementaryFigure 1. P2X7R activationcauses a time- and dose-dependent reduction in UPS activity in N2a cells butdoes not affect the cellular translation or transcription capacities, the proteasomalcatalytic subunit β2, or the total amount of proteasome nor for affecting thecell viability. Supplementary figure 2. P2X7R activation induces dephosphorylation of Akt and GSK3β kinases in N2acells but does not modify the cytosolic STAT3 levels. Supplementary figure 3. In vivo P2X7R activation reduces the β5 andβ1 but does not β2 mRNA levels. Astrocytes are not affected by P2X7R inducedUPS regulation. Supplementary figure 4. Human AD patients present a reduced hippocampal chymotrypsin-like activity, butnot post-glutamyl-like activity nor inducible β5i or β1i expression levels. Invivo P2X7R blockage reverts the decreased chymotrypsin-like andpost-glutamyl-like proteasomal activities and the number of hippocampal cellsbearing polyubiquitinated aggregates in P301S mice. [file 13195_2023_1258_MOESM1_ESM.zip › Supplementary material resubSR.docx]

**Supplementary Material**

**P2X7 inhibition ameliorates ubiquitin-proteasome system dysfunction associated with Alzheimer's disease.**

Carolina Bianchi^1, 2*^, Beatriz Alvarez-Castelao^1*^, Álvaro Sebastián-Serrano^1, 2^, Caterina Di Lauro^1, 2^, Lucia Soria Tobar^1, 2^, Annette Nicke^3^, Tobias Engel^4^ and Miguel Díaz-Hernández^1, 2*^.

***Corresponding author**: Miguel Díaz Hernández

Postal address: Dpto. Bioquímica y Biología Molecular, Facultad de Veterinaria, Universidad Complutense de Madrid, Avenida Puerta de Hierro s/n, 28040 Madrid

Spain.

Tel.: +34-913944068

Fax: +34-913943909

Email: [migueldiaz@ucm.es](mailto:migueldiaz@ucm.es)


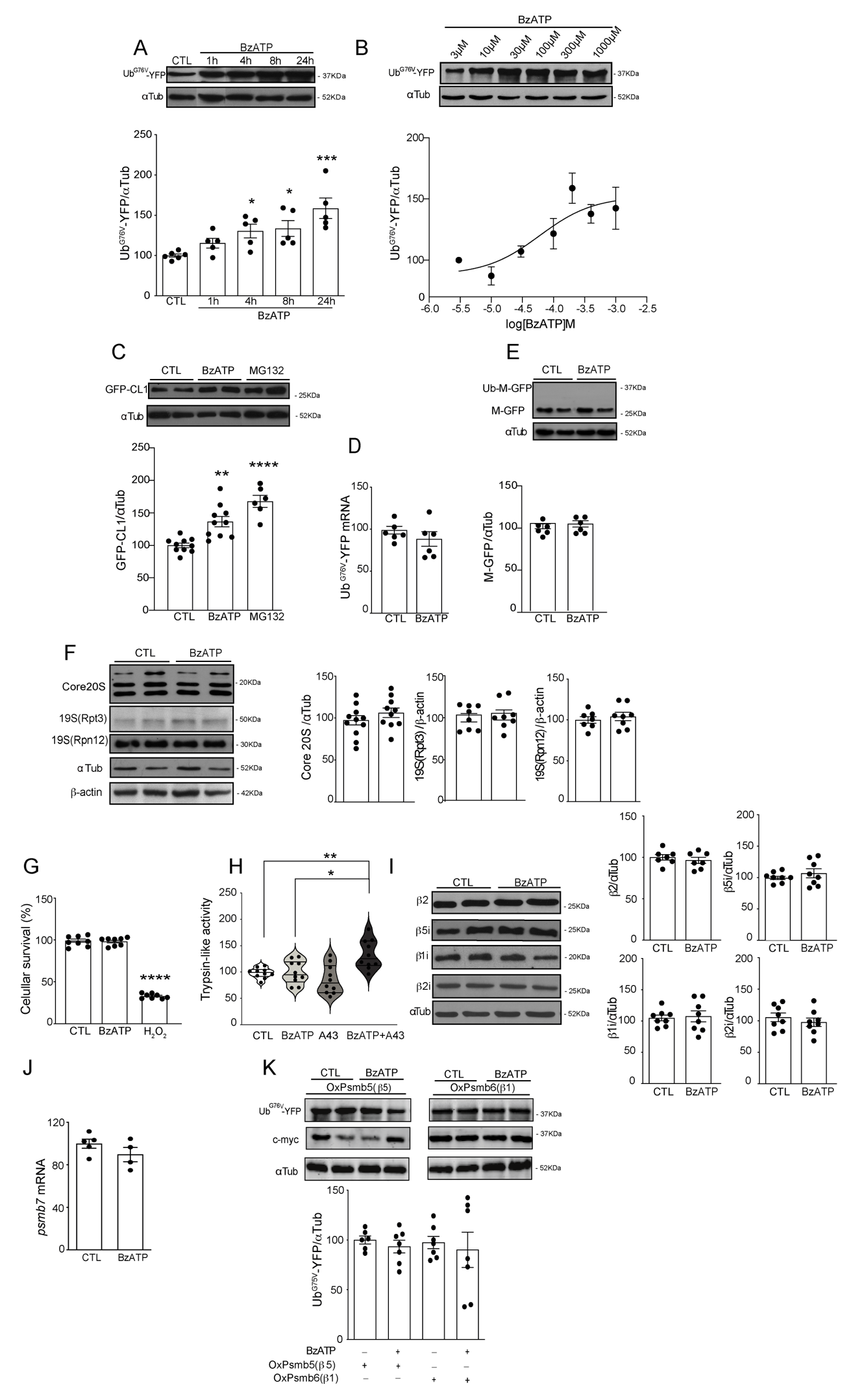


**Supplementary Figure 1: P2X7R activation causes a time- and dose-dependent reduction in UPS activity in N2a cells but does not affect the cellular translation or transcription capacities, the proteasomal catalytic subunit β2, or the total amount of proteasome nor for affecting the cell viability.** Western blot analysis of N2a cells expressing Ub^G76V^-YFP after being stimulated with the selective P2X7R agonist BzATP at different concentrations and timing. A) The addition of 200μM BzATP to N2a cells expressing Ub^G76V^-YFP for different stimulation periods (from 1 h to 48 h) enhanced the basal levels of Ub^G76V^-YFP (n ≥ 5 in triplicate). B) Dose–response curve obtained treating N2a cells expressing Ub^G76V^-YFP for 24 h with BzATP at different concentrations (n ≥ 3 in triplicate). C) Stimulation of N2a cells expressing GFP-CL1 with 200 μM BzATP for 24 h also enhanced the basal levels of UPS reporter (n ≥6 in triplicate). P2X7R overexpression in Ub^G76V^-YFP expressing N2A cells increases the UPS reporter accumulation induced by 200μM BzATP. D) Analysis of Ub^G76V^-YFP mRNA levels by quantitative RT-PCR in cells treated or not with BzATP for 24 h (n = 6 in triplicate). E) Western blot analysis of N2a cells expressing Ub-M-GFP after being stimulated with vehicle solution or 200 μM BzATP. The band corresponding to Ub-M-GFP protein was not detected by Western blot and the quantification of GFP protein levels did not show significant differences between both treatments. 100% corresponded to the Ub^G76V^-YFP, GFP-CL1 or Ub-M-GFP protein expression detected in cells treated with vehicle solution. F) Expression levels of the proteasome components 19S and 20S in cells treated or not with 200μM BzATP for 24 h (n ≥ 7 in triplicate). Membranes were probed with anti-α-tubulin antibody to correct any possible deviation on protein loading. 100% corresponds to the proteasome subunits expression detected in cells treated with vehicle solution. G) Treatment with 200 μM BzATP for 24 h did not significantly modify the viability of cells (F, n = 5 in triplicate). H) Measurement of trypsin-like peptidase activity assayed in N2a cells stimulated with 200μM BzATP in presence or absence of 40μM A438079 (n=3 in triplicated). 100% value corresponds to activity detected in vehicle-treated N2a cells. In all reactions, the specificity of the fluorogenic reaction was verified by addition of the MG132 proteasome inhibitor. I) Western blot analysis of β2, β5i, β1i, β2i proteasome subunits expression in N2a cells stimulated with 200 BzATP or vehicle solution (n ≥ 8). J) Quantitative PCR analysis of β2 (*Psmb7*) mRNA levels in vehicle-(CTL) or BzATP- treated N2a cells. Values were normalized concerning the internal GAPDH control (n≥ 4 in duplicated). K) Representative Western blot image and quantification of Ub^G76V^-YFP transfected N2a cells overexpressing β5 or β1 and stimulated with 200μM BzATP or vehicle for 24 hours. Graphs show the quantification of Ub^G76V^-YFP levels. Data in bar graphs represent mean ± s.e.m. *p ≤ 0.05, **p ≤ 0.01 and ***p ≤ 0.001; ***p ≤ 0.0001 using a one-way ANOVA followed by Tukey’s post hoc test.


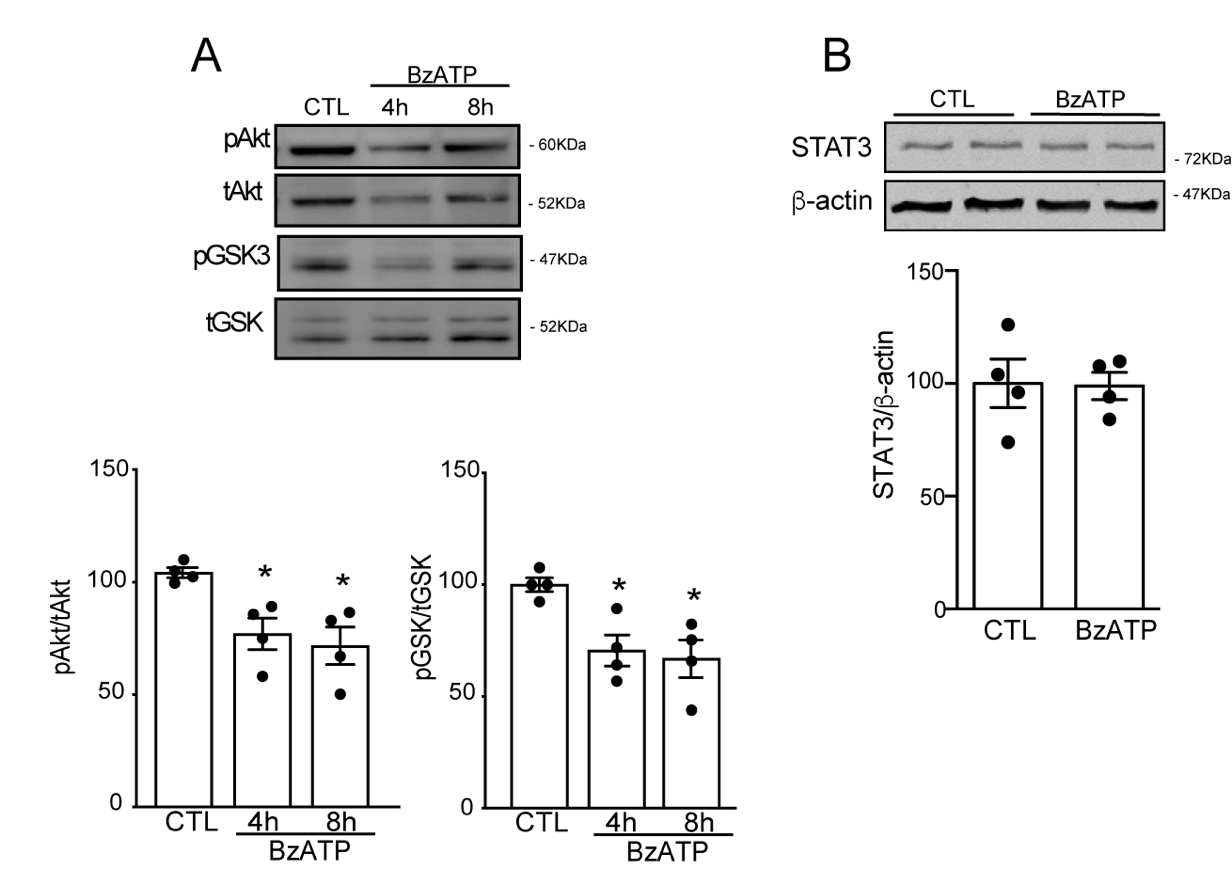


**Supplementary figure 2: P2X7R activation induces dephosphorylation of Akt and GSK3β kinases in N2a cells but does not modify the cytosolic STAT3 levels.**

A) Western blot analysis of pAkt, total Akt, GSK3β or GSK3β levels in N2a cells stimulated with 200 BzATP or vehicle solution for 4 or 8h. Graphs show 200μM BzATP increase phosphorylation levels of Akt and GSK3β in a time dependent way (n = 4 in duplicate). Membranes were normalized with anti-total Akt or anti total GSK3β antibodies for normalization propose. B) Representative Western blot image and quantification of STAT3 levels in N2a cells stimulated with 200μM BzATP or vehicle for 24 hours. Graphs show the quantification of cytosolic STAT3 levels. Data in bar graphs represent mean ± s.e.m. *p ≤ 0.05, using a one-way ANOVA followed by Tukey’s post hoc test.


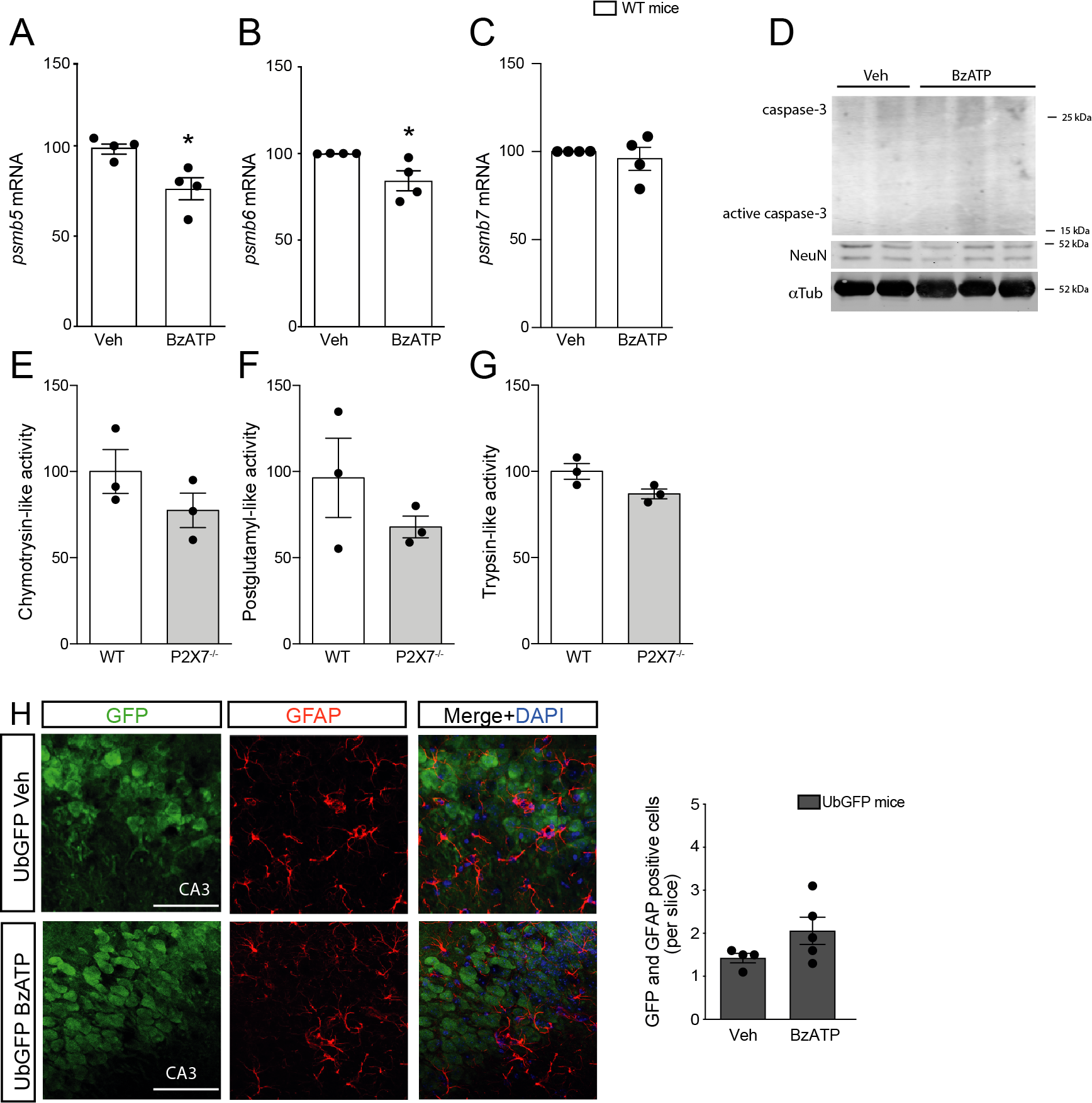


**Supplementary figure 3, *In vivo* P2X7R activation reduces the β5 and β1 but does not β2 mRNA levels. Astrocytes are not affected by P2X7R induced UPS regulation.**

Quantitative PCR analysis of β5 (*Psmb5*, A) and β1 (*Psmb6*, B), and β2 (*Psmb7*, C) mRNA levels in the ipsilateral hippocampus of WT mice treated with a single injection of 2μL 30 mM BzATP or vehicle solution (Veh) via i.c.v. for 24 h (n = 4 mice per group). D) Representative western blot analysis to measure apoptotic cell death using activated caspase-3 antibody and neuronal survival using NeuN antibody in WT mice i.c.v. treated with Veh or BzATP 24 hours. Sample loading was normalized with anti-α-tubulin (α-Tub) antibody. E-G) Chymotrypsin-like (E), postglutamyl (F), and Trypsin-like (G) activities were measured in hippocampal extracts from WT mice (white bars) or P2X7R^-/-^ mice (grey bars) (n≥ 3 in duplicated). 100% value corresponds to activity detected in WT mice. In all reactions, the specificity of the fluorogenic reaction was verified by addition of the proteasome inhibitor MG132. H) Hippocampal sections from BzATP- or vehicle-treated UbGFP mice stained with specific astroglial marker (GFAP, red channel) and GFP (green channel). Merged images showing DAPI channel in blue are also showed. Scale bar: 100 μm. Quantification of the number of GFP positive cells identified as astroglia cells per hippocampal slice in BzATP- or vehicle-treated UbGFP mice (n ≥ 5 mice per treatment and n≥ 3 sections per mouse). Data in bar graphs depict means ± s.e.m. *p≤0.05; using a using an unpaired two tailed Student’s t-test.


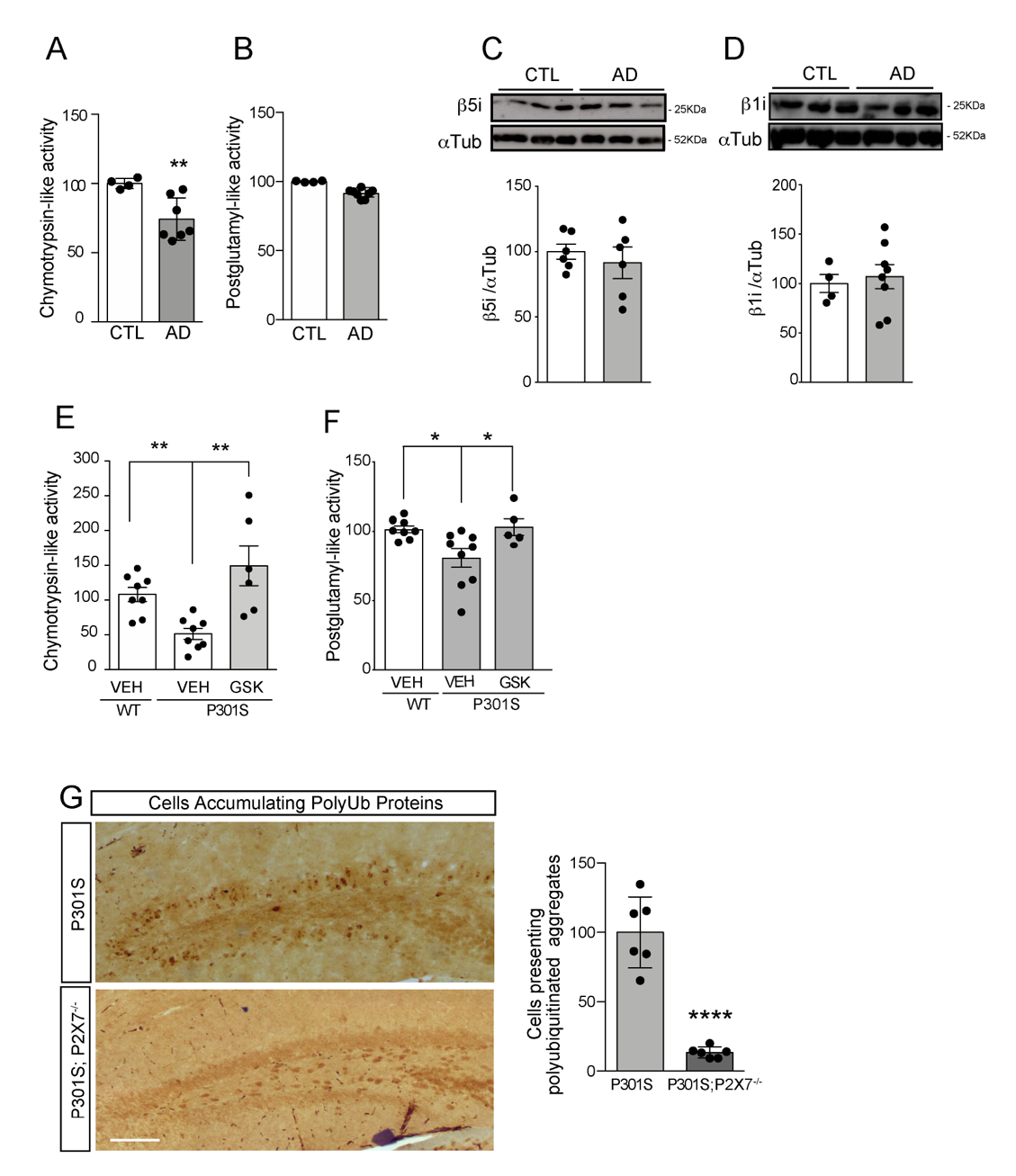


**Supplementary figure 4; Human AD patients present a reduced hippocampal chymotrypsin-like activity, but not post-glutamyl-like activity nor inducible β5i or β1i expression levels. *In vivo* P2X7R blockage reverts the decreased chymotrypsin-like and post-glutamyl-like proteasomal activities and the number of hippocampal cells bearing polyubiquitinated aggregates in P301S mice.** Graphs shows chymotrypsin-like (A) and postglutamil-like (B) activities measured in hippocampal extracts from AD patients (n = 7) and non-affected individuals (CTL) (n = 4). Western blot and quantification of β5i (C) and β1i (D) protein levels in homogenates from hippocampal AD patients (n = 8) and control individuals (n = 4). In all cases, the 100 % value corresponds to the averaged amount of β5i and β1i protein level detected in the hippocampi of non-affected individuals. Graphs shows chymotrypsin-like (E) and postglutamil-like (F) activities measured in hippocampal extracts from vehicle-treated WT mice (Veh), vehicle- treated P301S and GSK-treated P301S (n≥4 in triplicate per genotype and condition). G) Representative images of hippocampal slices from P301S mice and P301S;P2X7^-/-^ mice stained with FK2 antibody. Scale bar: 100 μm. Graph shows the number of hippocampal cells bearing intracellular polyubiquitinated aggreges in P301S and P301S;P2X7^-/-^ mice (n=6 per genotype) . Data in bar graphs represent mean ± s.e.m. * P≤ 0.05 ; ** P ≤ 0.01;**** P ≤ 0.0001 using an unpaired two-tailed Student’s t-test.
