# Supplementary material for: P2X7 receptor inhibition ameliorates ubiquitin–proteasome system dysfunction associated with Alzheimer’s disease
Source: Alzheimers Res Ther. 2023 Jun 7;15:105. doi: 10.1186/s13195-023-01258-x (PMC10245610; doi:10.1186/s13195-023-01258-x)

Suppl. Figure 1A

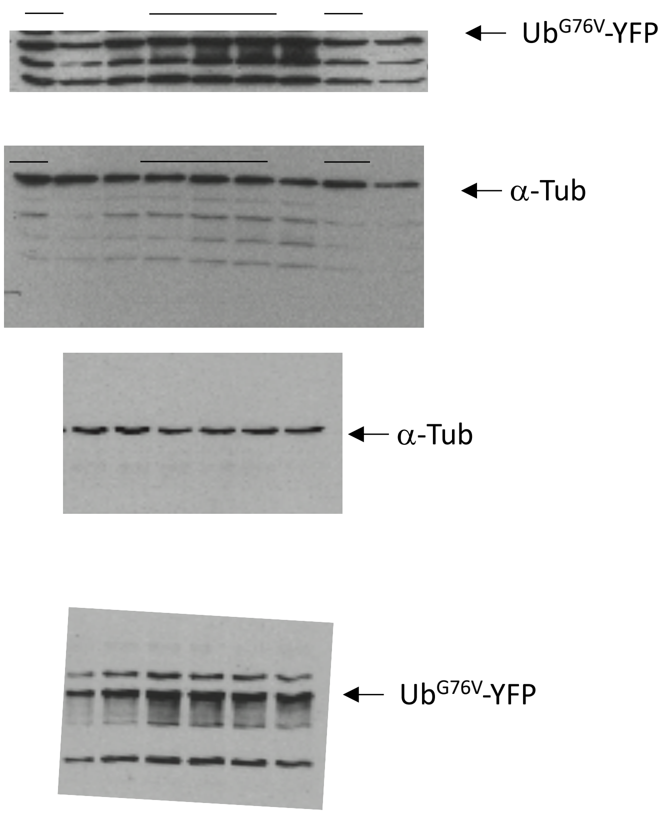

Suppl. Figure 1C

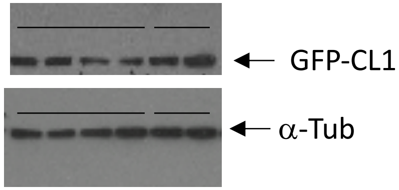

Suppl. Figure 1E

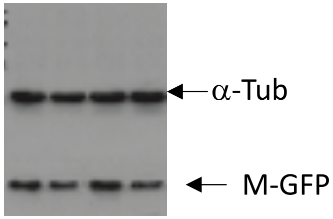

Suppl. Figure 1F

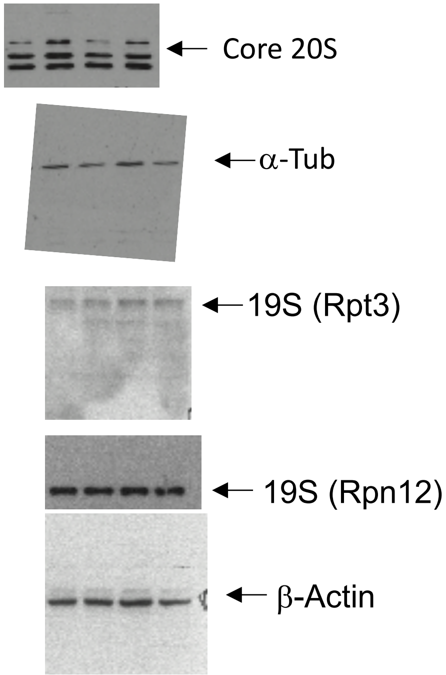

Suppl. Figure 1I

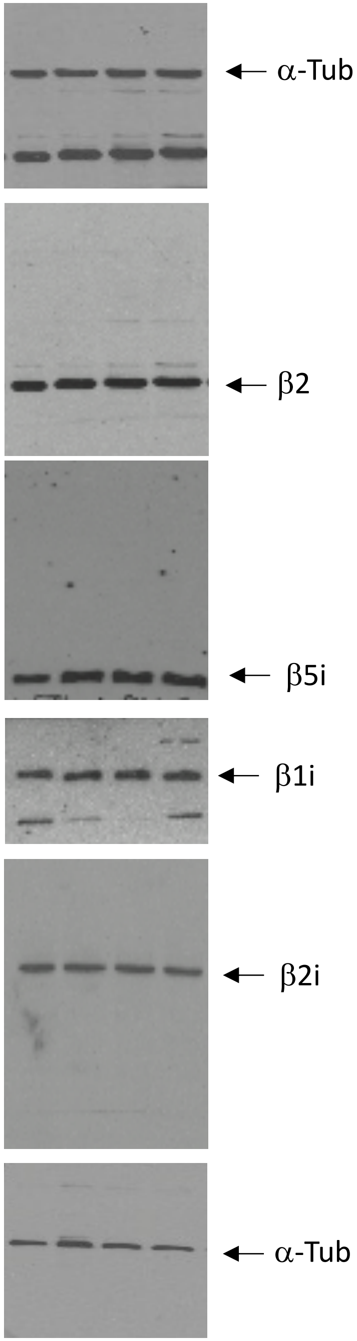

Suppl. Figure 1K

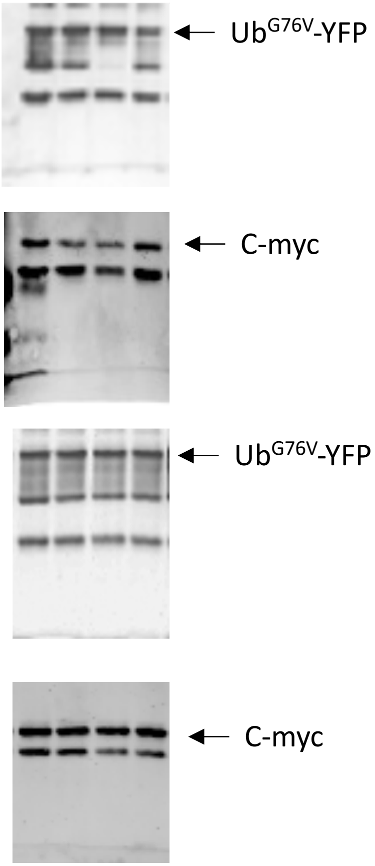

Suppl. Figure 1I

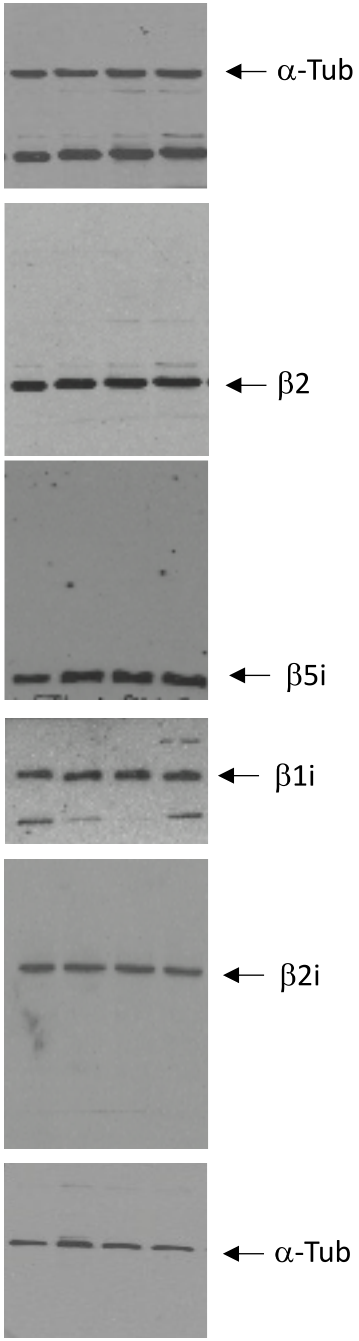

Suppl. Figure 1K

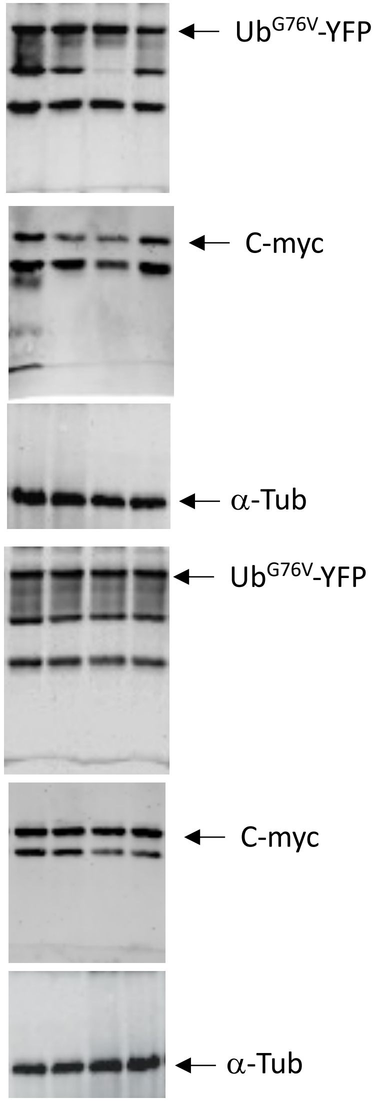

Suppl. Figure 2A

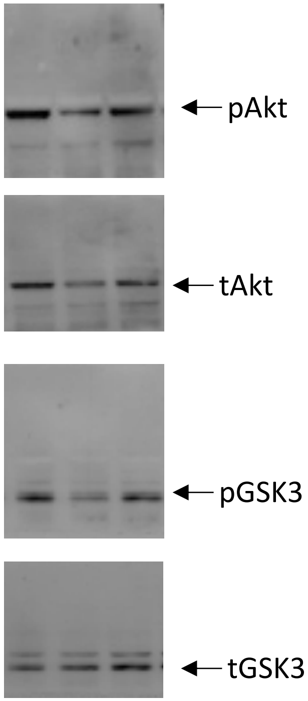

Suppl. Figure 2B

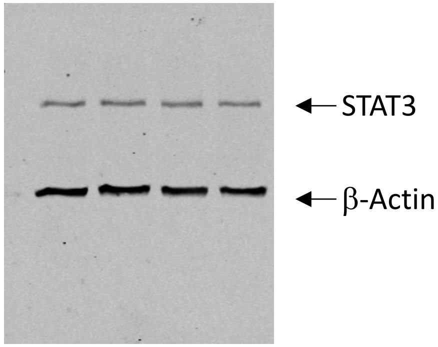

Suppl. Figure 3D

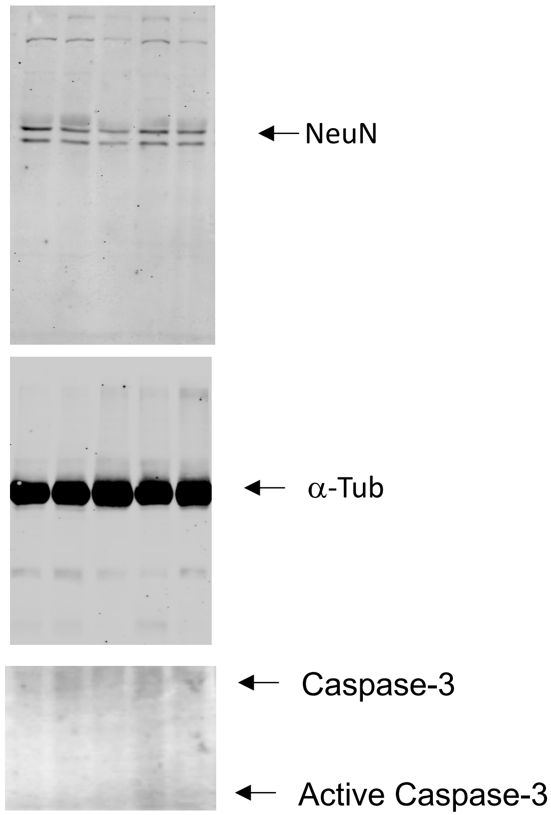

Suppl. Figure 4C

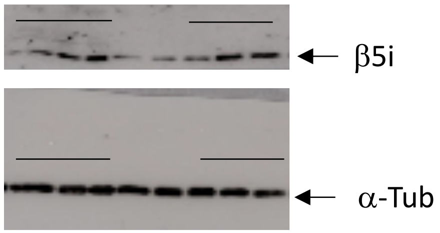

Suppl. Figure 4D

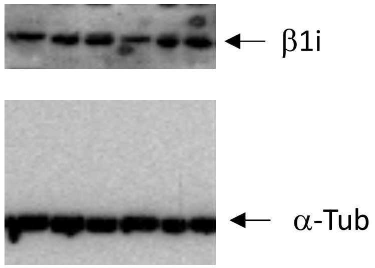

Supplement: Supplementary file 1 — Additional file 1: SupplementaryFigure 1. P2X7R activationcauses a time- and dose-dependent reduction in UPS activity in N2a cells butdoes not affect the cellular translation or transcription capacities, the proteasomalcatalytic subunit β2, or the total amount of proteasome nor for affecting thecell viability. Supplementary figure 2. P2X7R activation induces dephosphorylation of Akt and GSK3β kinases in N2acells but does not modify the cytosolic STAT3 levels. Supplementary figure 3. In vivo P2X7R activation reduces the β5 andβ1 but does not β2 mRNA levels. Astrocytes are not affected by P2X7R inducedUPS regulation. Supplementary figure 4. Human AD patients present a reduced hippocampal chymotrypsin-like activity, butnot post-glutamyl-like activity nor inducible β5i or β1i expression levels. Invivo P2X7R blockage reverts the decreased chymotrypsin-like andpost-glutamyl-like proteasomal activities and the number of hippocampal cellsbearing polyubiquitinated aggregates in P301S mice. [file 13195_2023_1258_MOESM1_ESM.zip › WBs supplementary figures.pdf]
