# Supplementary material for: P2X7 receptor inhibition ameliorates ubiquitin–proteasome system dysfunction associated with Alzheimer’s disease
Source: Alzheimers Res Ther. 2023 Jun 7;15:105. doi: 10.1186/s13195-023-01258-x (PMC10245610; doi:10.1186/s13195-023-01258-x)
Supplement: Supplementary file 2 — Additional file 2: Supplementary Table 1. Information of Control, Alzheimer’s, and Pick’s disease cases analyzed [file 13195_2023_1258_MOESM2_ESM.docx]

**TABLES**

| **Diagnosis** | **Sex** | **Age**  **(years)** | **Western**  **Blot analysis** | **Quantitative Real-Time PCR** |
| --- | --- | --- | --- | --- |
| Alzheimer  Alzheimer  Alzheimer  Alzheimer  Alzheimer  Alzheimer  Alzheimer  Alzheimer  Control  Control  Control  Control  Control  Control | M  M  M  M  M  F  F  F  F  F  F  F  M  F | 76  69  68  63  57  94  86  65  83  74  61  58  73  70 | √  √  √  √  √  √  √  √  √  √  √  √  √  √ | √  √  √  √  √  √  √  √  √  √  √  √  √ |

M, male. F, female. All samples are hippocampus.

Supplementary Table 1. Information of Control and Alzheimer´s disease cases analyzed
